# KIF13B Attenuates Sepsis-Induced Myocardial Dysfunction through the Stabilization of PLIN5

**DOI:** 10.34133/research.1033

**Published:** 2026-01-12

**Authors:** Lianxin Zhang, Guolin Miao, Si Mei, Yufei Han, Yitong Xu, Wenxi Zhang, Jingxuan Chen, Kaikai Lu, Yinqi Zhao, Zihao Zhou, Jinxuan Chen, Jiabao Guo, Pingping Lai, Sin Man Lam, Guanghou Shui, Ling Zhang, Weiguang Zhang, Wei Huang, Yuhui Wang, Xunde Xian

**Affiliations:** ^1^ Institute of Cardiovascular Sciences, State Key Laboratory of Vascular Homeostasis and Remodeling, School of Basic Medical Sciences, Peking University, Beijing, China.; ^2^ Department of Cardiology and Institute of Vascular Medicine, Peking University Third Hospital, Beijing, China.; ^3^ State Key Laboratory of Molecular Developmental Biology, Institute of Genetics and Developmental Biology, Chinese Academy of Sciences, Beijing, China.; ^4^ Lipidall Technologies Company Limited, Changzhou 213022, Jiangsu Province, China.; ^5^ Department of Human Anatomy and Histology and Embryology, School of Basic Medical Sciences, Peking University, Beijing, China.; ^6^ Beijing Key Laboratory of Cardiovascular Receptors Research, Peking University Third Hospital, Beijing, China.

## Abstract

Sepsis-induced cardiac dysfunction (SICD) is a major contributor to mortality in sepsis. Kinesin family member 13B (KIF13B) has been identified as a critical protective factor for metabolic disorder and cardiovascular disease; however, the role of KIF13B in SICD remains unknown. After introducing lipopolysaccharide or cecal ligation and puncture surgery to wild-type (*WT*) and global *Kif13b* knockout (*Kif13b*^−/−^) mice combined with lipopolysaccharide-treated neonatal rat cardiomyocytes, we found that KIF13B expression levels were markedly down-regulated in septic hearts and cardiomyocytes. *Kif13b* deletion exacerbated SICD progress with reduced cardiac contractile function and resulted in increased mortality, accompanied by promoted lipid accumulation, fibrosis, and mitochondrial impairment. Mechanistically, the loss of KIF13B enhanced the lysosomal degradation of the lipid-droplet-associated protein perilipin 5 (PLIN5), thus disrupting the mitochondrial localization of PLIN5 and then impairing cardiac lipid homeostasis and proper mitochondrial function. Nevertheless, cardiac-directed AAV9-*PLIN5* gene therapy sufficiently corrected cardiac dysfunction, inhibited lipid accumulation, and reduced oxidative stress in *Kif13b*^−/−^ mice with SICD. In summary, these findings provide a new insight into the molecular mechanism underlying the pathogenesis of SICD, highlighting the KIF13B/PLIN5 axis as a potential therapeutic target for the treatment of SICD.

## Introduction

Sepsis, a life-threatening organ dysfunction triggered by a dysregulated host response to infection, remains a leading cause of global mortality. Despite important advances in supportive care and antimicrobial therapy, sepsis management faces substantial challenges. Sepsis-induced cardiac dysfunction (SICD), a common and severe complication, significantly increases mortality, emerging as a critical determinant of poor prognosis, with mortality rates reaching 70% to 90% [1,2]. The intricate pathophysiology of SICD involves a complex interplay of systemic inflammation, calcium handling abnormalities, mitochondrial impairment, and profound disruptions in cardiac energy metabolism and lipid homeostasis [3].

SICD specifically refers to the acute impairment of myocardial contractility and relaxation occurring during severe sepsis or septic shock, independent of ischemia or preexisting heart disease [3]. While inflammatory cascades and oxidative stress are well-established contributors, the precise mechanisms driving SICD are incompletely understood, posing important challenges for targeted therapeutic development. Current management primarily focuses on hemodynamic support and treating the underlying infection, lacking specific interventions to directly protect the myocardium [4]. A crucial limitation lies in the insufficient mechanistic detail regarding how sepsis alters fundamental cardiac processes, particularly energy substrate utilization [3]. Mounting evidence highlights a pivotal role for abnormal lipid metabolism within the heart during sepsis [5,6]. Dysregulation of fatty acid (FA) uptake, oxidation, and storage (lipotoxicity) significantly contributes to myocardial stunning and dysfunction [7], yet the specific molecular regulators orchestrating these metabolic shifts remain poorly defined, necessitating deeper investigation into the metabolic underpinnings of SICD.

Kinesin family member 13B (KIF13B), also known as guanylate kinase-associated kinesin, is the largest member of the kinesin-3 subfamily. It participates in diverse cellular functions, including organelle transport, inflammatory response, and neuronal development [8–10]. Intriguingly, KIF13B has been reported to act as a key regulator of cellular trafficking and metabolic processes [11–14]. Recently, emerging lines of evidence demonstrated that KIF13B facilitated the transport of lipid transporters and receptors, profoundly influencing cellular lipid uptake, synthesis, and cholesterol efflux [12,14]. Its deficiency or dysfunction predisposes experimental animals to hepatic steatosis, dyslipidemia, and accelerated atherosclerosis and abdominal aortic aneurysm [15–17], underscoring its central role in maintaining lipid balance. Given the vital dependence of cardiac function on precise lipid metabolic regulation and the established link between lipid dysregulation and SICD, we postulated that KIF13B might play a previously unrecognized role in protecting the heart during sepsis. However, direct evidence linking KIF13B to cardiac lipid metabolism or its potential function in mitigating SICD was entirely unexplored, demanding detailed investigation into its cardiac-specific actions during septic stress.

In the present study, we subjected *Kif13b* knockout (*Kif13b*^−/−^) mice and their wild-type (*WT*) littermates to well-established SICD models: lipopolysaccharide (LPS) challenge and cecal ligation and puncture (CLP). We demonstrate that KIF13B deficiency exacerbates SICD, manifested by a decreased cardiac ejection fraction (EF) and increased cardiac lipid accumulation, consequently reducing survival rates in SICD mice. Mechanistically, KIF13B interacts with the lipid droplet (LD) protein perilipin 5 (PLIN5), reducing its degradation in lysosomes, which enhances LD–mitochondrion contact, thereby alleviating the LPS-induced suppression of mitochondrial respiration and lipid accumulation. Ultimately, we employed adeno-associated virus serotype 9 (AAV9) to mediate cardiac-specific overexpression of *PLIN5* in *Kif13b*^−/−^ mice. This intervention mitigated SICD and improved the lipid accumulation phenotype. Collectively, this work provides the first evidence of KIF13B’s role in regulating cardiac lipid metabolism in SICD, offering novel theoretical foundations and potential therapeutic targets for preventing and treating SICD.

## Results

### KIF13B is down-regulated in the heart with SICD

**Fig. 1.**
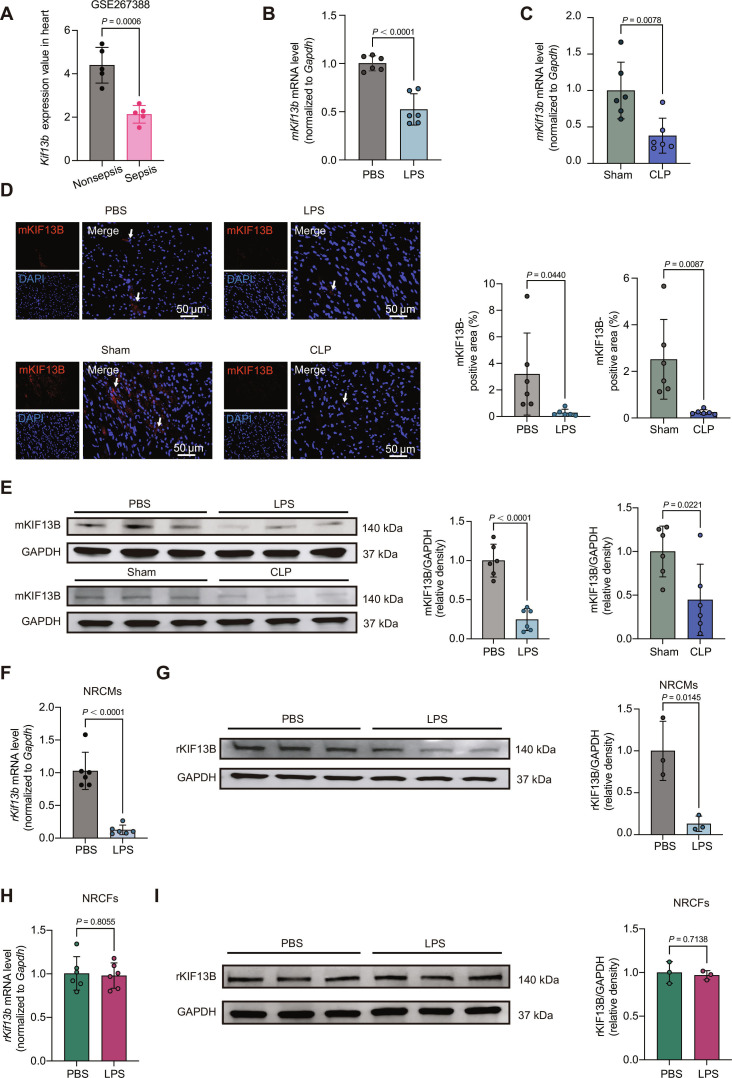
Kinesin family member 13B (KIF13B) expression is suppressed in the heart during sepsis-induced cardiac dysfunction. (A) *Kif13b* messenger RNA (mRNA) expression in mice with nonsepsis (*n* = 11) and sepsis from a Gene Expression Omnibus (GEO) dataset (GSE267388). (B and C) Quantitative real-time polymerase chain reaction (qPCR) analysis of *mKif13b* mRNA expression in sepsis mouse hearts (*n* = 6/group). (D) Immunofluorescent staining and quantitative analysis of mouse KIF13B (mKIF13B) in heart tissues from male wild-type (*WT*) mice (*n* = 6/group). (E) Protein abundances of mKIF13B in nonfailing heart tissues and sepsis-induced failing heart tissues from male *WT* mice (*n* = 6/group). (F) qPCR analysis of *rKif13b* mRNA expression in neonatal rat cardiomyocytes (NRCMs) (*n* = 3/group). (G) Protein abundances of rat KIF13B (rKIF13B) in NRCMs (*n* = 3/group). (H) qPCR analysis of *rKif13b* mRNA expression in neonatal rat fibroblasts (NRCFs) (*n* = 3/group). (I) Protein abundances of rKIF13B in NRCFs (*n* = 3/group). Data are presented as mean ± SD and analyzed by an unpaired Student *t* test in (A) to (I). PBS, phosphate-buffered saline; LPS, lipopolysaccharide; CLP, cecal ligation and puncture; DAPI, 4′,6-diamidino-2-phenylindole; GAPDH, glyceraldehyde-3-phosphate dehydrogenase.

To investigate the association between KIF13B and SICD, we analyzed *Kif13b* messenger RNA (mRNA) expression in the hearts nonseptic and septic mice using the GSE267388 database. The *Kif13b* mRNA levels were significantly down-regulated in septic mouse hearts (Fig. 1A). Subsequently, we established SICD mouse models via intraperitoneal injection of LPS or CLP surgery. Both models were validated by the characteristics of elevated lactate dehydrogenase (LDH) levels and impaired cardiac contractile function (Fig. S1). Quantitative real-time polymerase chain reaction (qPCR) confirmed an overt reduction in *Kif13b* mRNA in both LPS- and CLP-induced SICD models (Fig. 1B and C). Consistently, immunofluorescence staining and Western blotting revealed decreased KIF13B protein abundance in the SICD hearts as well (Fig. 1D and E).

As cardiomyocytes and fibroblasts are the main cells in the heart, to identify the alteration of KIF13B in these 2 cell types in the context of SCID, we stimulated neonatal rat cardiomyocytes (NRCMs) and neonatal rat fibroblasts (NRCFs) with LPS. The data of qPCR and Western blotting showed that LPS treatment overtly decreased *Kif13b* mRNA and protein expression levels in NRCMs, but not in NRCFs (Fig. 1F to I). These findings collectively indicate that KIF13B expression is visibly suppressed in the hearts of mice with SICD.

### The loss of *Kif13b* exacerbates cardiac dysfunction and lipid accumulation

**Fig. 2.**
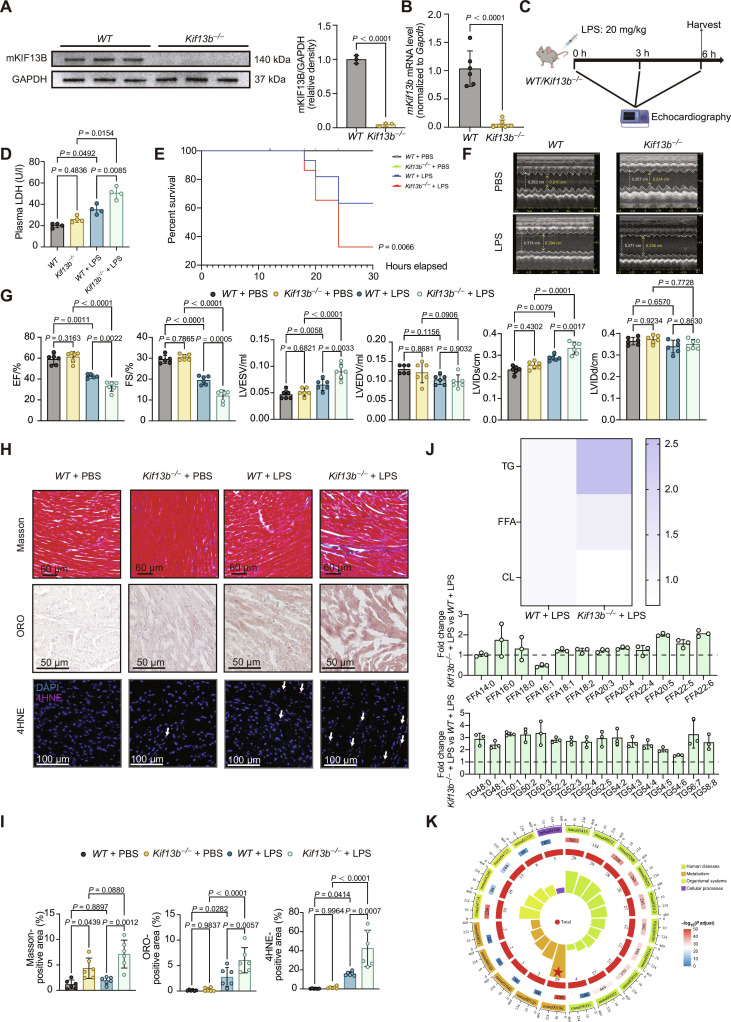
KIF13B deficiency exacerbates sepsis-induced cardiac dysfunction by disrupting myocardial lipid metabolism; 8- to 10-week-old male *Kif13b*^−/−^ mice were injected with PBS or LPS (20 mg/kg) (*n* = 6/group). (A) Protein abundances of mKIF13B in heart tissues from male *WT* and *Kif13b*^−/−^ mice. (B) qPCR analysis of *mKif13b* mRNA expression in heart tissues from *WT* or *Kif13b*^−/−^ mice (*n* = 6/group). (C) Schematic diagram of mouse experiments. (D) Quantitative analysis of plasma lactate dehydrogenase (LDH) from mice. (E) Survival curves of *WT* and *Kif13b*^−/−^ mice injected with LPS (*n* = 10/group). (F) Representative echocardiography M-mode images obtained from the indicated mice in (C) at 6 h after LPS administration. (G) ejection fraction (EF), fractional shortening (FS), left ventricular end-systolic volume (LVESV), left ventricular end-diastolic volume (LVEDV), left ventricular internal diameter at end-systole (LVIDs), and left ventricular internal diameter at end-diastole (LVIDd) were quantified via echocardiography (*n* = 5 to 6/group). (H) Representative images of Masson, Oil Red O (ORO), and 4-hydroxynonenal (4HNE) staining of heart sections from the indicated mice in (C). Scale bars, 60 μm for Masson staining, 50 μm for ORO staining, and 100 μm for 4HNE staining. (I) Quantitative analysis of the percentage of the fibrotic area, ORO-positive area, and 4HNE-positive area in relation to the total area (*n* = 6/group). (J) Three significantly increased lipid species in the heart analyzed by the targeted lipidome (*n* = 3/group). (K) Kyoto Encyclopedia of Genes and Genomes (KEGG) pathway analysis of differentially expressed genes based on the RNA sequencing data of the heart tissues from male *WT* and *Kif13b*^−/−^ mice injected with LPS (20 mg/kg) (*n* = 4/group). Data are presented as mean ± SD and analyzed by an unpaired Student *t* test in (A) and (B); 2-way analysis of variance (ANOVA) in (D), (G), and (I); and log-rank (Mantel–Cox) test in (E). TG, triglyceride; FFA, free fatty acid; CL, cardiolipin.

To determine KIF13B’s role in the development of SICD, we generated global *Kif13b* knockout (*Kif13b*^−/−^) mice, and both qPCR and Western blotting confirmed complete *Kif13b* deletion in heart tissue (Fig. 2A and B), respectively. Afterward, LPS was administered intraperitoneally to 8- to 10-week-old male wild-type (*WT*) and *Kif13b*^−/−^ mice to induce SICD (Fig. 2C). Compared to *WT* mice, LPS-treated *Kif13b*^−/−^ mice exhibited an increase in LDH levels with a decreased survival ratio (Fig. 2D and E). Moreover, *Kif13b* deficiency caused important cardiac dysfunction at 6 h post-LPS stimulation, evidenced by reduced EF and fractional shortening (FS) and increased left ventricular end-systolic volume (LVESV) and left ventricular internal diameter at end-systole (LVIDs), with no alteration in left ventricular end-diastolic volume and left ventricular internal diameter at end-diastole (Fig. 2F and G), while the loss of *Kif13b* did not significantly affect the heart weight or left ventricular cavity under basal or septic conditions (Fig. S2A and B). Histological analysis revealed increased myocardial fibrosis, lipid accumulation, and oxidative stress in septic *Kif13b*^−/−^ mice versus *WT* mice (Fig. 2H and I). Lipidomics analysis indicated elevated triglyceride (TG) and free fatty acids (FFAs) and reduced cardiolipin (CL) in *Kif13b*^−/−^ mice (Fig. 2J). Kyoto Encyclopedia of Genes and Genomes enrichment analysis of differentially expressed genes down-regulated in cardiac transcriptomes revealed that *Kif13b* deficiency markedly impaired oxidative phosphorylation (OXPHOS) pathways (Fig. 2K). Similar exacerbation of plasma LDH, cardiac dysfunction, fibrosis, lipid accumulation, and oxidative stress were observed in the CLP model using *Kif13b*^−/−^ mice (Fig. S3). These results demonstrate that *Kif13b* ablation exacerbates SICD, aligning with observed metabolic pathway dysregulation.

### *Kif13b* knockdown impairs cardiomyocyte lipid metabolism and mitochondrial function upon LPS stimulation

**Fig. 3.**
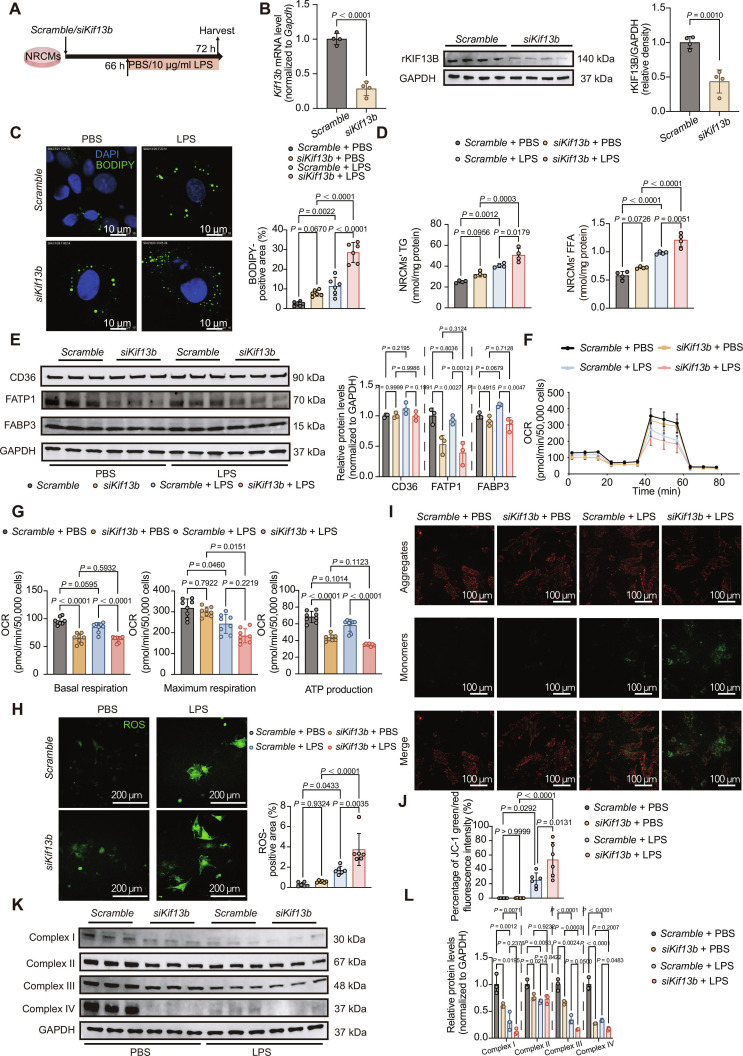
The loss of KIF13B disrupts lipid metabolism and the mitochondrial electron transfer chain function in cardiomyocytes under sepsis stress. (A) Schematic diagram of in vitro sepsis-induced cardiomyocyte dysfunction model construction. (B) Left: qPCR analysis of *rKif13b* mRNA expression in cultured NRCMs. Right: Protein abundances of rKIF13B in NRCMs transfected with *Scramble* or *siKif13b* (*n* = 4/group). (C) BODIPY staining (green) of cultured NRCMs with 10 μg/ml LPS or PBS and quantitative analysis of the BODIPY-positive area in the total area (*n* = 6/group). (D) Quantitative analysis of NRCMs’ FFA and TG (*n* = 4/group). (E) Protein abundances of CD36, fatty acid transport protein 1 (FATP1), and fatty acid binding protein 3 (FABP3) NRCMs (*n* = 3/group). (F) Oxygen consumption rate (OCR) of NRCMs during the sequential addition of oligomycin, carbonyl cyanide 4-(trifluoromethoxy)phenylhydrazone (FCCP), and antimycin A, as indicated in (A). (G) The bar charts of mitochondrial respiration changes in NRCMs, which were analyzed with basal respiration, maximal respiration, and adenosine triphosphate (ATP) production (*n* = 8/group). (H) Reactive oxygen species (ROS) staining (green) of cultured NRCMs with 10 μg/ml LPS or PBS and quantitative analysis of the ROS-positive area in the total area (*n* = 6/group). (I and J) NRCMs stained with 5 μM JC-1. Cells were excited at 555 or 488 nm and imaged by a confocal microscope. Scale bars, 100 μm for JC-1 staining (*n* = 6/group). (K and L) Protein abundances of mitochondrial complexes in NRCMs (*n* = 3/group). Data are presented as mean ± SD and analyzed by 2-way ANOVA in (C) to (E), (G), (H), (J), and (L) and an unpaired Student *t* test in (B).

Given similar cardiac phenotypes in LPS and CLP models, subsequent mechanistic studies used the LPS-induced in vitro sepsis model (Fig. 3A). First, we knocked down *Kif13b* in NRCMs followed by LPS (10 μg/ml) stimulation. qPCR and Western blotting confirmed efficient *Kif13b* knockdown (Fig. 3B). BODIPY staining revealed LPS-induced LD accumulation in the cardiomyocytes, and cardiomyocytes with *Kif13b* silencing displayed smaller but more numerous droplets (Fig. 3C). Lipid extraction showed increased TG and FFA in KIF13B-knockdown cardiomyocytes after LPS treatment (Fig. 3D). The net imbalance between lipid intake and consumption can promote lipid accumulation within cardiomyocytes. To investigate whether this imbalance results from altered lipid uptake, we first examined the expression levels of key receptor proteins involved in lipid import and found no change in CD36 protein levels. Since previous evidence indicates that inhibition of CD36 palmitoylation disrupts FA uptake [18], we further assessed the palmitoylation status of CD36. Our results showed that KIF13B knockdown did not affect CD36 palmitoylation under either basal or LPS-stimulated conditions (Fig. S4). Thus, we conclude that KIF13B does not modulate cardiac lipid uptake through changes in CD36 signaling pathway.

Moreover, we also determined the expression levels of fatty acid transport protein 1 (FATP1) and fatty acid binding protein 3 (FABP3), the 2 key lipid transport mediators in heart. We observed that FATP1 levels were decreased following KIF13B knockdown under both phosphate-buffered saline (PBS) and LPS conditions, while FABP3 expression remained unchanged in PBS-treated cells but was down-regulated in LPS-stimulated cells with KIF13B knockdown (Fig. 3E). These findings further suggest that lipid accumulation is unlikely to be driven by enhanced uptake in the setting of KIF13B deficiency.

Given that cardiomyocytes largely rely on lipids for mitochondrial energy production, we next assessed mitochondrial OXPHOS using Seahorse XF assays in NRCMs. KIF13B knockdown significantly impaired basal respiration and adenosine triphosphate (ATP) production. In contrast, the maximum respiratory capacity was reduced only upon LPS stimulation (Fig. 3F and G), indicating that silencing *Kif13b* leads to substantial mitochondrial dysfunction.

To explore whether this mitochondrial impairment stems from defects in lipid metabolic pathways, we examined the mRNA expression of key enzymes responsible for FA activation to acyl-CoA in hearts from both *WT* and *Kif13b*^−/−^ mice. Our analysis revealed that the mRNA levels of these enzymes were comparable or elevated across genotypes (Fig. S5A), suggesting that KIF13B deficiency does not impair this initial step of FA metabolism. However, metabolomic profiling revealed important accumulation of acylcarnitine, an intermediate metabolite generated from acyl-CoA prior to β-oxidation (Fig. S5B). Consistent with a defect in mitochondrial substrate utilization, KIF13B-knockdown NRCMs exhibited elevated reactive oxygen species (ROS), reduced mitochondrial membrane potential (ΔΨm) (Fig. 3H to J), and impaired electron transport chain (ETC) complex expression (Fig. 3K and L). Together, these results indicate that mitochondrial dysfunction in *Kif13b*-deficient cardiomyocytes arises primarily from direct damage to mitochondrial integrity and function, rather than from upstream defects in lipid uptake or activation.

### KIF13B overexpression attenuates LPS-induced lipid accumulation and mitochondrial dysfunction in cardiomyocytes

**Fig. 4.**
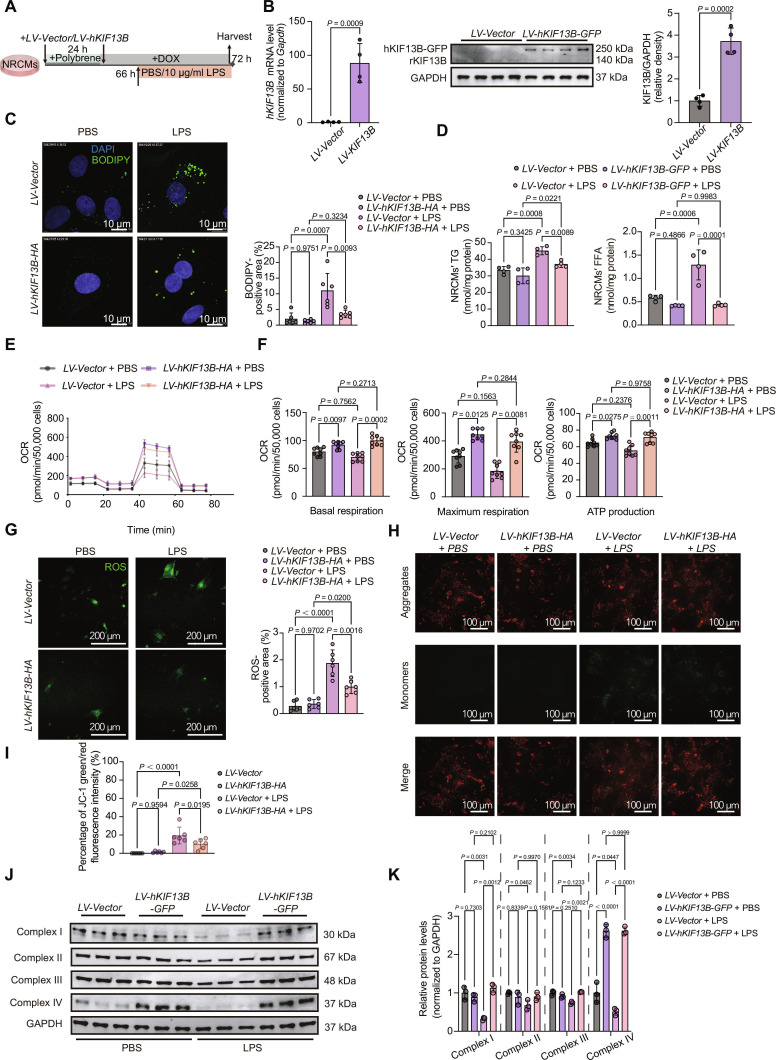
Cardiomyocyte KIF13B overexpression mitigates sepsis-induced cardiac dysfunction by enhancing lipid clearance and mitochondrial respiration. (A) Schematic diagram of in vitro sepsis-induced cardiomyocyte dysfunction model construction. (B) Left: qPCR analysis of *hKIF13B* mRNA expression in cultured NRCMs. Right: Protein abundances of KIF13B (including rKIF13B and human KIF13B [hKIF13B]) in NRCMs infected with *LV-Vector* or *LV-hKIF13B-GFP* (*n* = 4/group). (The sequence identities among human, mouse, and rat KIF13B exceeded 80%. Therefore, the sequence common to human, mouse, and rat was utilized to design antibodies.) (C) BODIPY staining (green) of cultured NRCMs with 10 μg/ml LPS or PBS and quantitative analysis of the BODIPY-positive area in the total area (*n* = 6/group). (D) Quantitative analysis of NRCMs’ FFA and TG (*n* = 4/group). (E) OCR of NRCMs during sequential addition of oligomycin, FCCP, and antimycin A, as indicated in (A). (F) The bar charts of mitochondrial respiration changes in NRCMs, which were analyzed with basal respiration, maximal respiration, and ATP production (*n* = 8/group). (G) ROS staining (green) of cultured NRCMs with 10 μg/ml LPS or PBS and quantitative analysis of the ROS-positive area in the total area (*n* = 6/group). (H and I) NRCMs stained with 5 μM JC-1. Cells were excited at 555 or 488 nm and imaged by a confocal microscope. Scale bars, 100 μm for JC-1 staining (*n* = 6/group). (J and K) Protein abundances of mitochondrial complexes in NRCMs (*n* = 3/group). Data are presented as mean ± SD and analyzed by 2-way ANOVA in (C), (D), (F), (G), (I), and (K) and an unpaired Student *t* test in (B). DOX, doxorubicin; HA, hemagglutinin; GFP, green fluorescent protein.

Subsequently, we overexpressed human KIF13B in NRCMs followed by LPS stimulation (Fig. 4A). qPCR and Western blotting confirmed an increase in both the mRNA and protein levels of KIF13B (Fig. 4B). Cells overexpressing KIF13B showed reduced formation of LPS-induced LDs (Fig. 4C) with decreased TG and FFA contents (Fig. 4D). Seahorse XF assays demonstrated that KIF13B overexpression enhanced the basal and maximum respiration capacities and ATP production (Fig. 4E and F), indicating improved mitochondrial function. Meanwhile, ROS generation was reduced and ΔΨm was restored in cardiomyocytes with KIF13B overexpression after LPS stimulation (Fig. 4G to I). Notably, overexpression of *KIF13B* effectively reversed the damage of mitochondrial ETC in NRCMs (Fig. 4J and K). Taken together, these results demonstrate that KIF13B negatively regulates LPS-induced lipid accumulation and maintains mitochondrial function in cardiomyocytes.

### KIF13B protects cardiomyocytes from sepsis-induced lipotoxicity and mitochondrial dysfunction by upregulating PLIN5

**Fig. 5.**
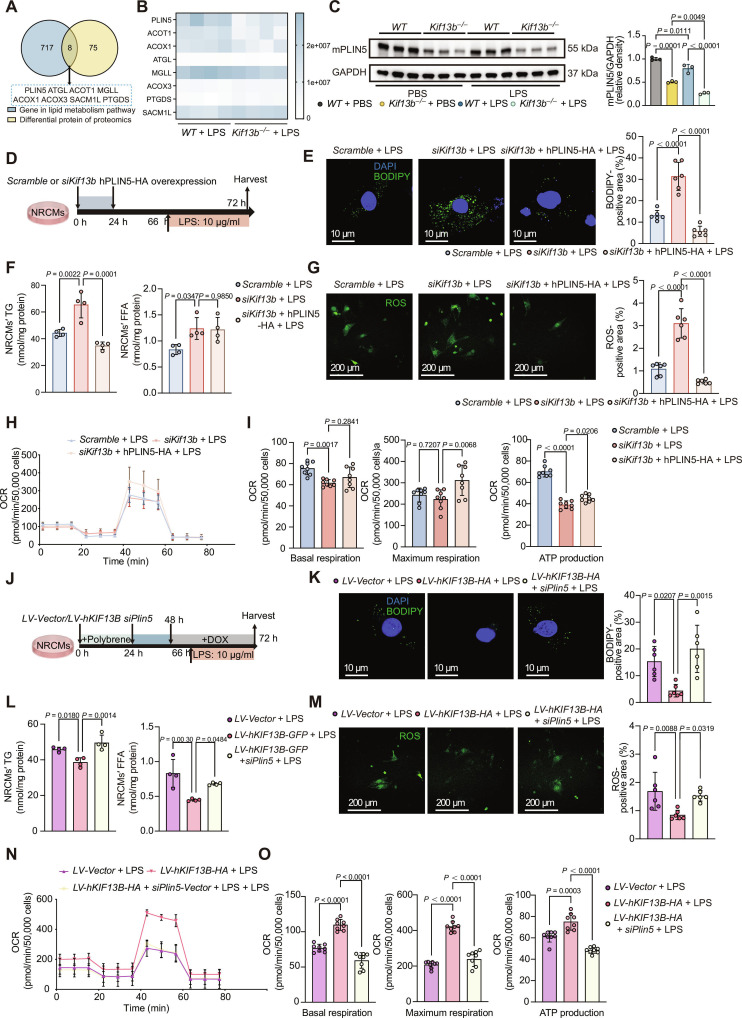
Perilipin 5 (PLIN5) mediates KIF13B-dependent protection from lipotoxicity and mitochondrial dysfunction in sepsis-stressed cardiomyocytes. (A) Venn diagram of heart differentially expressed proteins from proteomics and lipid metabolism pathway genes. (B) Heat map of differentially expressed proteins in (A). (C) Protein abundances of PLIN5 in heart tissues from male *WT* or *Kif13b*^−/−^ mice (*n* = 3/group). (D) Schematic diagram of in vitro sepsis-induced cardiomyocyte dysfunction model construction. (E) BODIPY staining (green) of cultured NRCMs with 10 μg/ml LPS and quantitative analysis of the BODIPY-positive area in the total area (*n* = 6/group). (F) Quantitative analysis of NRCMs’ FFA and TG (*n* = 4/group). (G) ROS staining (green) of cultured NRCMs with 10 μg/ml LPS and quantitative analysis of the ROS-positive area in the total area (*n* = 6/group). (H) OCR of NRCMs during sequential addition of oligomycin, FCCP, and antimycin A, as indicated in (D). (I) The bar charts of mitochondrial respiration changes in NRCMs, which were analyzed with basal respiration, ATP production, and maximal respiration (*n* = 8/group). (J) Schematic diagram of in vitro sepsis-induced cardiomyocyte dysfunction model construction. (K) BODIPY staining (green) of cultured NRCMs with 10 μg/ml LPS and quantitative analysis of the BODIPY-positive area in the total area (*n* = 6/group). (L) Quantitative analysis of NRCMs’ FFA and TG (*n* = 4/group). (M) ROS staining (green) of cultured NRCMs with 10 μg/ml LPS and quantitative analysis of the ROS-positive area in the total area (*n* = 6/group). (N) OCR of NRCMs during sequential addition of oligomycin, FCCP, and antimycin A, as indicated in (J). (O) The bar charts of mitochondrial respiration changes in NRCMs, which were analyzed with basal respiration, maximal respiration, and ATP production (*n* = 8/group). Data are presented as mean ± SD and analyzed by 2-way ANOVA in (C) and 1-way ANOVA in (E) to (G), (I), (K) to (M), and (O). mPLIN5, mouse PLIN5; hPLIN5, human PLIN5.

To elucidate how cardiomyocyte-derived KIF13B governed SICD progression, we conducted an integrated analysis of lipid metabolism pathway genes and differentially expressed proteins from the hearts of SICD *Kif13b*^−/−^ mice versus *WT* mice, in which PLIN5 showed the most important difference (Fig. 5A and B). Western blotting confirmed observably reduced PLIN5 protein in the hearts of *Kif13b*^−/−^ mice (Fig. 5C). Similarly, the level of PLIN5 protein exhibited a comparable reduction in NRCMs transfected with *siKif13b* (Fig. S6A). Restoring PLIN5 in NRCMs with KIF13B knockdown (Fig. 5D) obviously reduced lipid accumulation, TG levels, and ROS production but rescued ΔΨm (Fig. 5E to G and Fig. S6B). Seahorse XF assays indicated that the knockdown of KIF13B led to a decrease in the basal respiratory capacity and the restoration of PLIN5 did not change this situation (Fig. 5H and I). However, PLIN5 supplementation restored the maximum respiratory capacity and ATP production (Fig. 5H and I).

Conversely, PLIN5 knockdown in the setting of overexpressing *KIF13B* increased lipid accumulation, TG, FFA, and ROS but reduced ΔΨm compared with *KIF13B* overexpression alone (Fig. 5J to M and Fig. S6C). Seahorse XF assays confirmed that overexpression of KIF13B restored the basal respiratory capacity, maximum respiratory capacity, and ATP production of cardiomyocytes, but this protective effect was abolished after PLIN5 knockdown (Fig. 5N and O). These data suggest that KIF13B protects against sepsis-induced lipotoxicity and mitochondrial dysfunction primarily by regulating the lipid-droplet-associated protein PLIN5 in cardiomyocytes.

### KIF13B orchestrates PLIN5 trafficking from lysosomal degradation to maintain lipid homeostasis

**Fig. 6.**
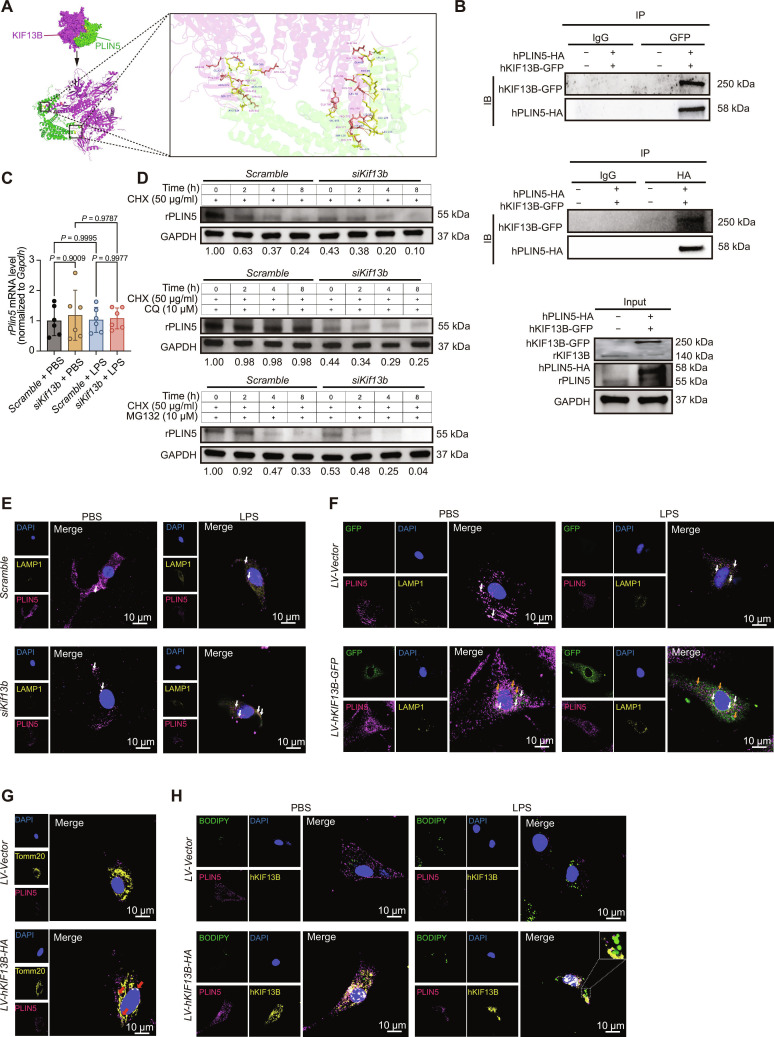
KIF13B governs PLIN5 stability and localization to couple lipid droplets with mitochondrial metabolism. (A) Left: Surface representation of the HDOCK-predicted complex, with KIF13B shown in purple and PLIN5 shown in green. The predicted binding interface is highlighted within the dashed box. Right: Close-up view of the predicted binding interface (boxed region at left). Key interacting residues are shown as sticks. Putative hydrogen bonds are indicated by orange dashed lines. The structural model was generated using the HDOCK server (http://hdock.phys.hust.edu.cn/). Visualization and analysis were performed using PyMOL (version 2.4). (B) Co-immunoprecipitation (Co-IP) analysis of the interaction between KIF13B and PLIN5 in NRCMs transfected with hKIF13B-GFP and hPLIN5-HA. (C) qPCR analysis of *Plin5* mRNA expression in NRCMs transfected with *Scramble* and *siKif13b* (*n* = 6/group). (D) Western blotting of rat PLIN5 (rPLIN5) in NRCMs transfected with *Scramble* and *siKif13b* treated with PBS, 10 μM MG132, or 10 μM chloroquine (CQ) in the presence of 50 μg/ml cycloheximide (CHX) for the indicated time points. (E) Immunofluorescent staining of lysosome-associated membrane protein 1 (LAMP1; yellow) and PLIN5 (purple) in NRCMs transfected with *Scramble* or *siKif13b*. (F) Immunofluorescent staining of LAMP1 (yellow), PLIN5 (purple), and GFP (green) in NRCMs infected with *LV-Vector* or *LV-hKIF13B-GFP*. (G) Immunofluorescent staining of Tomm20 (yellow) and PLIN5 (purple) in NRCMs infected with *LV-Vector* or *LV-hKIF13B-HA*. (H) Immunofluorescent staining of hKIF13B (yellow), PLIN5 (purple), and BODIPY (green) in NRCMs infected with *LV-Vector* or *LV-hKIF13B-HA*. Data are presented as mean ± SD and analyzed by 2-way ANOVA. IgG, immunoglobulin G; IP, immunoprecipitation; IB, immunoblotting.

To explore how KIF13B impacted PLIN5 expression, we predicted a high probability of KIF13B–PLIN5 interaction using the HDOCK server (Fig. 6A), which was confirmed by co-immunoprecipitation (Fig. 6B). However, the mRNA level of *Plin5* gene remained unchanged after the knockdown of KIF13B (Fig. 6C), suggesting that KIF13B probably regulated PLIN5 expression at the posttranslational level. Using cycloheximide to inhibit protein synthesis combined with the lysosomal inhibitor chloroquine or the proteasomal inhibitor MG132, we found that PLIN5 degradation was inhibited only by the co-treatment of cycloheximide and chloroquine (Fig. 6D), indicating that KIF13B stabilized PLIN5 by inhibiting its lysosomal degradation. Co-staining PLIN5 with the lysosomal marker lysosome-associated membrane protein 1 (LAMP1) showed reduced PLIN5 signal after KIF13B knockdown, with LPS increasing PLIN5–LAMP1 co-localization (Fig. 6E). Conversely, KIF13B overexpression elevated PLIN5 protein levels and diminished its co-localization with the lysosomal marker LAMP1 (Fig. 6F). Meanwhile, KIF13B also significantly enhanced the co-localization of PLIN5 with Tomm20-labeled mitochondria (Fig. 6G). Notably, KIF13B itself was found to co-localize with PLIN5 on LDs as well (Fig. 6H). Together, these findings support a model wherein KIF13B binds to PLIN5 in the cytoplasm, enhances its stability by suppressing lysosomal degradation, and facilitates its trafficking to both LDs and mitochondria, thereby enabling PLIN5 to exert its physiological roles.

### Cardiac-directed delivery of *PLIN5* reverses sepsis-associated heart failure in *Kif13b*^−/−^ mice

**Fig. 7.**
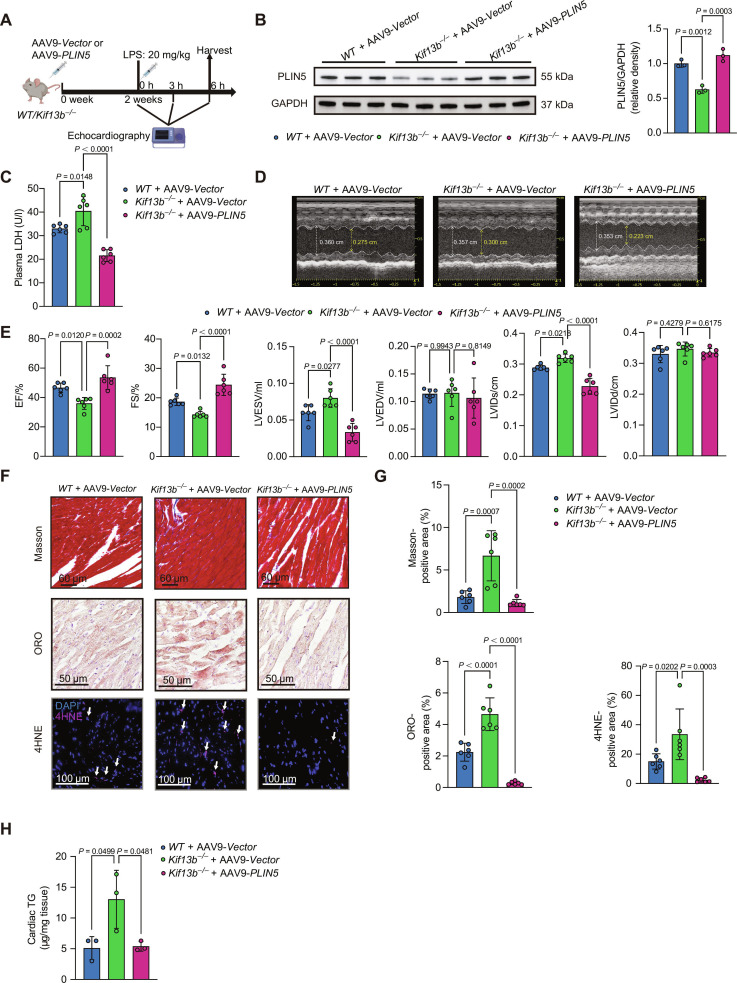
Cardiac-specific restoration of PLIN5 mitigates sepsis-induced cardiac dysfunction in *Kif13b*^−/−^ mice. (A) Schematic diagram of mouse experiments. (B) Protein abundances of PLIN5 in heart tissues from male *WT* or *Kif13b*^−/−^ mice injected with AAV9-*Vector* or AAV9-*PLIN5* (*n* = 3/group). AAV9, adeno-associated virus serotype 9. (C) Quantitative analysis of plasma LDH from mice. (D) Representative echocardiography M-mode images obtained from the indicated mice in (A) at 6 h after LPS administration. (E) EF, FS, LVESV, LVEDV, LVIDs, and LVIDd were quantified via echocardiography (*n* = 6/group). (F and G) Representative images and quantitative analysis of Masson, ORO, and 4HNE staining of heart sections from the indicated mice in (A). Scale bars, 60 μm for Masson staining, 50 μm for ORO staining, and 100 μm for 4HNE (*n* = 6/group). (H) Heart TG content (*n* = 3/group). Data are presented as mean ± SD and analyzed by one-way ANOVA.

Finally, in order to evaluate whether PLIN5 is a therapeutic target for KIF13B-deficiency-associated SICD, we restored *PLIN5* in the hearts of *Kif13b*^−/−^ mice using AAV9 with *cTNT* promoter (Fig. 7A). Western blotting confirmed cardiac *PLIN5* restoration in the hearts of *Kif13b*^−/−^ mice (Fig. 7B). We found that the supplementation of PLIN5 notably reduced the serum LDH level in septic *Kif13b*^−/−^ mice and restored cardiac function by increasing EF and FS and decreasing the LVESV and LVIDs (Fig. 7C to E). Consistent with improved cardiac function, restoring PLIN5 in *Kif13b*^−/−^ mice attenuated myocardial fibrosis and lipid accumulation and more profoundly, reduced oxidative stress relative to that in *Kif13b*^−/−^ controls (Fig. 7F to H). These findings demonstrate that cardiac-specific *PLIN5* overexpression mitigates SICD in *Kif13b*^−/−^ mice, highlighting its therapeutic potential for SICD.

## Discussion

**Fig. 8.**
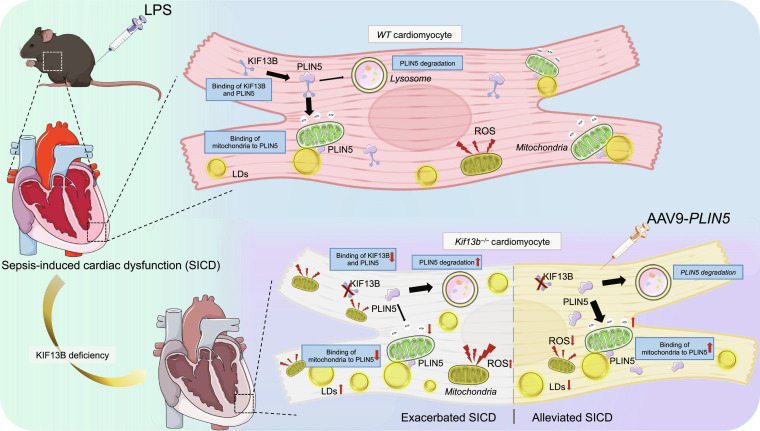
Schematic representation of the cardioprotective role of the KIF13B/PLIN5 axis in sepsis-induced cardiac dysfunction (SICD). In SICD, the deficiency of KIF13B reduces PLIN5 abundance in cardiomyocytes. This leads to lipid accumulation, mitochondrial dysfunction, and elevated ROS production. However, PLIN5 supplementation rescues the exacerbated cardiac dysfunction caused by KIF13B deficiency. Mechanically, KIF13B stabilizes PLIN5 in cardiomyocytes, preventing its lysosomal degradation and facilitating its mitochondrial localization. This process enhances lipid utilization, boosts ATP production, and mitigates lipid toxicity associated with ectopic lipid deposition. LDs, lipid droplets.

SICD is a prevalent and severe complication that significantly increases mortality. It has emerged as a crucial determinant of poor prognosis for sepsis. In septic cardiomyopathy, lipid metabolism disorders, such as the inability to convert FAs into acyl-CoA, along with intrinsic mitochondrial impairment and dynamic imbalance, collectively result in a myocardial energy crisis and a decline in contractile function. The 2 form a vicious cycle through inflammation, ROS, and metabolic reprogramming. Therefore, a deeper understanding of the precise molecular mechanisms underpinning the development of SICD is essential for the development of treatments for this devastating human disease. In the present study, we revealed that the KIF13B/PLIN5 axis serves as an important regulatory machinery in cardiac lipid homeostasis during sepsis. *Kif13b*^−/−^ mice subjected to sepsis exhibited exacerbated cardiac dysfunction, increased mortality, heightened fibrosis, pronounced lipid accumulation, and enhanced oxidative stress. Furthermore, cardiac-targeted *PLIN5* delivery significantly ameliorated these pathological phenotypes in septic *Kif13b*^−/−^ mice, improving cardiac function (Fig. 8). These findings demonstrate that the down-regulation of endogenous KIF13B is a important pathogenic driver, not merely an epiphenomenon, in the development of septic cardiomyopathy.

Previous studies indicated that KIF13B, the largest member of the kinesin superfamily, was conventionally recognized for microtubule-dependent transport of vesicles and organelles [11,19,20]. Recent investigations conducted in our laboratory have demonstrated that in metabolic dysfunction associated fatty liver disease, KIF13B interacts with AMPKα1 to inhibit hepatic de novo lipogenesis and improve FA oxidation, thereby preventing the development of fatty liver disease [15], indicating that KIF13B plays an important role in lipid metabolism homeostasis. Based on the aforementioned findings in the liver, we reported for the first time that in the heart, KIF13B safeguards cardiomyocyte lipid homeostasis and mitochondrial function in the setting of sepsis by stabilizing PLIN5 protein and then regulating its localization, thereby controlling mitochondrial energy production. To our knowledge, lipid overload not only directly disrupts cellular structure but also induces mitochondrial oxidative stress and respiratory chain dysfunction, ultimately resulting in a myocardial energy crisis [21,22], perpetuating a vicious cycle of metabolic stress. Specifically, inactivation of *Kif13b* exacerbated lipid accumulation and impaired mitochondrial respiration in LPS-challenged cardiomyocytes, which could be rescued by PLIN5 supplementation; however, the protective function of KIF13B in SICD was abolished after silencing *Plin5*, supporting the concept that the role of KIF13B in cardiac lipid regulation and energy homeostasis is PLIN5 dependent. Consistently, other studies also demonstrated that genetic ablation of *Plin5* induced cardiac lipid dysregulation and elevated oxidative stress under physiological conditions [23,24]. Notably, *Plin5*-deficient hearts exhibited significantly reduced LDs and TG content. The consequent release of FFAs serves as substrates for mitochondrial energy production, leading to excessive OXPHOS. This hypermetabolic state generates damaging ROS, resulting in cellular injury [25,26]. Conversely, in marked contrast to the phenotypes caused by *Plin5* deficiency, *Kif13b* deletion also reduced PLIN5 protein levels, yet paradoxically led to increased LD accumulation, elevated TG content, and heightened FFA levels within cardiomyocytes. This observed difference may be attributed to the direct regulatory role of KIF13B in mitochondrial function. Specifically, depleting KIF13B significantly impairs the expression of subunits from mitochondrial complexes I, II, III, IV and compromises the mitochondrial ETC and membrane potential, which are further impaired by LPS stimulation. Consequently, the excess FFAs cannot be efficiently utilized in OXPHOS. Instead, these surplus FFAs accumulate intracellularly, triggering a compensatory program of TG synthesis as a protective storage mechanism. Importantly, the concurrent loss of PLIN5 and inadequate cellular energy supply further promoted the lipolysis of stored TG. This dysfunctional cycle, which impaired FFA oxidation driving TG storage, followed by futile lipolysis due to mitochondrial damage, failed to resolve the lipid overload.

PLIN5 is highly expressed in tissues with a high FA oxidation capacity, particularly in cardiac tissue. It orchestrates the interaction between LDs and mitochondria, playing a crucial role in energy metabolism and lipid storage [27,28]. Mechanistic insights of this study indicated that PLIN5, a key regulatory protein on the LD surface, acted as a core effector downstream of KIF13B. Previous studies have reported that PLIN5 expression can be transcriptionally upregulated through peroxisome proliferator-activated receptor alpha activation [29], and its metabolic activity is modulated via protein kinase A-mediated phosphorylation at Ser155 [30]. In the present study, we provide new evidence that KIF13B exerted dual regulatory functions on PLIN5 regulation. KIF13B interacted directly with PLIN5 in cardiomyocytes, thereby stabilizing its intracellular abundance by inhibiting lysosomal degradation, and subsequently facilitated PLIN5 translocation to LDs and mitochondria, where PLIN5 balanced FA availability for mitochondrial oxidation with sequestration to protect against cytotoxicity. The loss of KIF13B resulted in enhanced PLIN5 targeting to lysosomes, leading to a important reduction in PLIN5 protein levels. Through spatiotemporal control of transport, KIF13B regulated PLIN5 localization. The results of the immunofluorescence co-localization analysis demonstrated that the presence of KIF13B significantly enhanced the co-localization of PLIN5–LDs or PLIN5–mitochondria. This spatial proximity was a critical prerequisite for the formation of LD–mitochondrion contacts. These findings indicated a dependent metabolic regulatory axis in which KIF13B modulated lipid metabolic flux and energy conversion efficiency through PLIN5 stabilization and positioning. Furthermore, stabilizing PLIN5 levels enables controlled lipolysis of TG and prevents the acute release of lipotoxic FFA. KIF13B-driven co-localization of PLIN5–LDs and PLIN5–mitochondria drastically shortened the spatial distance for FFA transfer from the LD storage depot to the mitochondrial oxidation site. This facilitates the efficient, directed transport of FFA into mitochondria, where they serve as high-quality substrates for β-oxidation, driving ATP synthesis to meet the heart’s energy demands. Collectively, these elements formed a novel signaling axis that meticulously regulated lipid dynamics and energy supply. While this further broadens the application of kinesins in cellular metabolism, whether KIF13B’s microtubule-transport activity contributes to PLIN5 dynamics or organelle interactions remains an open question for future investigation.

In ordinary circumstances, cardiomyocytes require substantial energy to maintain contractile function, primarily fueled by FA oxidation, which generates 60% to 90% of cardiac ATP via OXPHOS [31,32]. This process relies on the ETC, oxygen, adenine nucleotide translocators, and other components within the mitochondrial inner membrane. The ETC, comprising complexes I to IV along with coenzyme Q and cytochrome c, facilitates electron transfer to establish the proton gradient essential for ATP production [33]. However, in the context of sepsis, this crucial process of lipid metabolism undergoes substantial disruption. FA oxidation was suppressed, leading to myocardial energy underutilization and consequent cardiac dysfunction [31,34]. Consistent with previous reports, our findings demonstrated that targeting *Kif13b* impaired the function of myocardial mitochondrial complexes I to IV and led to the accumulation of both TG and FFA. It should be noted that the protein expression levels of key receptors required for myocardial FA uptake, including CD36, FATP1, and FABP3, were unchanged or even down-regulated, indicating that lipid accumulation stemmed from intrinsic metabolic impairment, not increased uptake.

Interestingly, lipidomics revealed a important decrease in CL contents in the septic *Kif13b*^−/−^ animals. CL depletion has been known to disrupt mitochondrial structure and function, including dissociation of supercomplexes, loss of membrane potential (ΔΨm), and OXPHOS defects [35,36]. Similarly, *Kif13b* knockdown also impaired mitochondrial function, manifesting as reduced basal and maximal respiration, decreased ATP production, increased ROS accumulation, diminished ΔΨm, and reduced ETC complex expression, which was further exacerbated by LPS stimulation. Importantly, impaired mitochondrial function further suppressed FA oxidation, creating a vicious cycle of worsening lipid accumulation and bioenergetic failure [37,38], whereas restoration of *Plin5* restored maximal respiratory capacity, ATP synthesis, and ΔΨm but reduced ROS accumulation. These findings aligned with 2 independent reports by Gallardo-Montejano et al. [39] and Kien et al. [40], suggesting that PLIN5 enhanced myocardial mitochondrial maximal respiration and reduced ROS. This evidence strongly implicated that the KIF13B/PLIN5 axis played a pivotal role in maintaining the functional homeostasis of cardiomyocytes, primarily by regulating lipid mobilization and oxidation capacity in response to increased energy demands. While basal respiration might depend on other pathways, this axis was essential for adaptive energy production under stress.

Furthermore, KIF13B deficiency resulted in a decrease in the levels of mitochondrial complex proteins, whereas *Kif13b* overexpression restored complexes I to III to their levels observed in the wild type, and also led to a important increase in complex IV. Consequently, *KIF13B* overexpression enhanced basal and maximal respiration and ATP production. It has been demonstrated that increased complex IV levels generally improved the integrated efficiency of the ETC, facilitated the process of OXPHOS, and consequently boosted ATP generation [41]. Nevertheless, excessive OXPHOS activity can potentially lead to increased accumulation of ROS [42,43]. We observed no concomitant ROS increase, indicating that KIF13B-mediated complex IV upregulation enhances ETC efficiency within physiological limits, avoiding detrimental oxidative stress. It will be tempting for us to explore the precise molecular mechanism by which KIF13B governs the expression of mitochondrial complex in future study.

To date, current clinical management of SICD remains primarily directed at improving hemodynamics or controlling inflammation, but therapeutic strategies specifically addressing the underlying metabolic defects remain scarce [44]. Therefore, activating the cardiac KIF13B/PLIN5 axis may be a compelling candidate for further investigation, which could offer a dual benefit by mitigating lipotoxicity caused by lipid accumulation and restoring cardiomyocyte energy metabolism, thereby cooperatively alleviating SICD. Given the high similarities in the metabolic traits between hamster species and humans [45–48], it will be tempting for us to further confirm the similar observations in this humanlike animal model in future study. Of note, neither solely modulating inflammation nor merely restoring energy metabolism is likely sufficient for optimal SICD treatment. Future therapeutic approaches combining PLIN5 delivery or small-molecule agonists of KIF13B with anti-inflammatory strategies hold important potential as a synergistic and potentially superior strategy for managing SICD.

It is noteworthy that while this study identifies KIF13B as a novel protective factor in SICD and elucidates its fundamental role in preserving cardiac function by enhancing PLIN5 stability and regulating its spatial distribution alongside mitochondrial function, the underlying cause for the reduced expression of KIF13B in the SICD disease state remains unclear. Elucidating the molecular mechanism responsible for KIF13B down-regulation in SICD is crucial for developing targeted small-molecule therapeutics that modulate KIF13B expression for precise treatment of this condition. Importantly, previous studies have suggested that epigenetic regulation via DNA methylation is implicated in SICD pathogenesis [49]. In line with this report, we observed a marked elevation in the methylation level of KIF13B in peripheral blood samples from septic patients compared with those of healthy controls (data not shown), indicating that hypermethylation may contribute to the decreased expression of KIF13B in septic heart tissue. In future work, we will further investigate the causal link between hypermethylation and KIF13B down-regulation in the septic heart and also delineate the specific molecular mechanisms involved in the transcriptional regulation of KIF13B.

In summary, this study for the first time clarifies that KIF13B acts as a regulator of lipid metabolism homeostasis and mitochondrial function in cardiomyocytes through PLIN5, exerting a beneficial effect in SICD. This highlights the significance of KIF13B in maintaining cardiomyocyte homeostasis and suggests that targeting cardiomyocyte-derived KIF13B or its downstream target PLIN5 could be a promising therapeutic strategy for treating SICD.

## Materials and Methods

Detailed methods are provided below and in the Supplementary Methods in the Supplementary Materials for all procedures carried out in this study. Biological replicates are incorporated for all datasets in this study. The datasets that support the findings of the study are available from the corresponding author upon reasonable request (xianxunde@bjmu.edu.cn).

### Experimental animal

All animal experiments were performed according to the principle of experimental animal care (National Institutes of Health publication no. 85Y23, revised 1996) and were approved by the Laboratory Animal Ethics Committee of Peking University (LA2023460). Wild-type (*WT*) C57BL/6J mice and Sprague–Dawley rats were purchased from Vital River Laboratory Animal Technology Co., Ltd. (Beijing, China). Global *Kif13b* knockout (*Kif13b*^−/−^) mice were preserved in our laboratory using the CRISPR/Cas9 gene editing system, as previously described [15]. The mice were given free access to a standard chow diet and water and maintained in a specific-pathogen-free standard facility with controlled temperature and humidity in a 12-h shift of light–dark cycle.

### Sepsis mouse model

Male *WT* and *Kif13b*^−/−^ mice at the age of 8 to 10 weeks were intraperitoneally injected with 20 mg/kg LPS (*Escherichia coli* 0111:B4, L2630, Sigma-Aldrich) dissolved in sterile PBS. For the control group, an equal volume of sterile PBS was given to the animals. Continuous survival monitoring was conducted until the death of all mice. Specifically, mice were euthanized by inducing anesthesia via inhalation of 5% isoflurane (R510-22-10, ‌RWD Life Science Co., Ltd.) in an induction chamber, followed by cervical dislocation and thoracotomy. After thoracotomy, the heart was rapidly excised to be fixed in 4% formaldehyde solution and embedded in paraffin or the left ventricular tissue was collected, which was rapidly frozen in liquid nitrogen and then stored at −80 °C for further analysis.

For concordance validation relative to LPS induction, the CLP model of polymicrobial sepsis was employed. Briefly, male *WT* and *Kif13b*^−/−^ mice were anesthetized before the CLP. All surgical procedures were performed on mice anesthetized with isoflurane. First, anesthesia was induced with 2% isoflurane in an induction chamber. Then, anesthesia was maintained throughout the surgical procedures with 1.2% isoflurane delivered via an anesthesia mask. Anesthetized mice were subjected to midline laparotomy. The cecum was then ligated tightly with a 6.0 silk suture at its base below the ileocecal valve and punctured with a 7-gauge needle. Afterward, the cecum was gently exteriorized, and the fecal content was expressed through the puncture sites. After the cecum returned into the peritoneal cavity, the abdominal cavity was closed with 2 epithelium layers, followed by 50 ml/g saline injection subcutaneously for resuscitation. Sham-operated mice underwent identical surgical procedures without cecal ligation or puncture. Cardiac function was assessed via echocardiography for sepsis mice.

### AAV9 gene delivery

AAV9 was purchased from Genomeditech Co., Ltd. (Shanghai, China). Mice were infected with AAV9 encoding *PLIN5* (AAV9-*PLIN5*) or an empty vector (AAV9-*Vector*). Six-week-old male *WT* and *Kif13b*^−/−^ mice were injected with AAV9 through the tail vein (2 × 10^11^ particles/mouse) and then subjected to the indicated experiments after 2 weeks.

### Echocardiography

Echocardiography was performed using an animal echocardiogram machine (VINNO Corporation, Suzhou, China). Mice were anesthetized with isoflurane inhalation at a concentration of 2% in an induction chamber and maintained at 1% isoflurane via an anesthesia mask during image acquisition. M-mode images were obtained from the left ventricular short axis at the papillary muscle level to assess the cardiac function.

### Cell culture and treatment

NRCMs were isolated from 3-d-old Sprague–Dawley rats in a manner described previously. Briefly, neonatal rats were euthanized by CO_2_ asphyxiation in a sealed chamber, with continuous gas flow maintained until the cessation of breathing. After thoracotomy, the excised hearts were dissected into small pieces and underwent sequential enzymatic digestion cycles (6 min/cycle) in 5 ml of buffer (0.6 mg/ml collagenase II [LS004176, Worthington Biochemical Corporation‌] and 1 mg/ml pancreatin [T8150, Solarbio] dissolved in sterile PBS). NRCMs were cultured in Dulbecco’s modified Eagle’s medium supplemented with 10% FBS (FS301-02, TransGen, China), 1% penicillin–streptomycin and 1 M bromodeoxyuridine for 2 d. NRCMs were treated with or without LPS (10 μg/ml) for 6 h and then incubated at 37 °C for 6 h.

### Statistical analyses

Differences between the 2 groups were compared using an unpaired Student *t* test. Multiple group comparisons were made by 1- or 2-way analysis of variance. Survival curves were analyzed by the log-rank (Mantel–Cox) test. All statistical tests were performed using the GraphPad Prism 9 software. Data were considered important at *P* < 0.05.

### Translational perspective

Reduced cardiac KIF13B levels may serve as a biomarker for SICD.

Targeting the KIF13B/PLIN5 axis may address metabolic dysfunction in septic cardiomyopathy beyond conventional anti-inflammatory approaches.

Cardiac-targeted AAV9-*PLIN5* gene therapy effectively reverses SICD in preclinical murine models with KIF13B deficiency.

Developing pharmacological agents that can stabilize the interaction of KIF13B and PLIN5 may represent a potential therapeutic target for SICD.

## Data Availability

The methods for this study’s procedures are outlined in the text and Supplementary Materials. This study includes biological replicates for its datasets, which are available upon request from the corresponding author.

## Supplementary Materials

Supplementary 1 Supplementary Methods Figs. S1 to S6 Tables S1 to S3 Reference [50]
