# Supplementary material for: KIF13B Attenuates Sepsis-Induced Myocardial Dysfunction through the Stabilization of PLIN5
Source: Research (Wash D C). 2026 Jan 12;9:1033. doi: 10.34133/research.1033 (PMC12794201; doi:10.34133/research.1033)
Supplement: Supplementary 1 — Supplementary Methods Figs. S1 to S6 Tables S1 to S3 Reference [50] [file research.1033.f1.zip › Supplementary Material Revise.docx]

**KIF13B Attenuates Sepsis-Induced Myocardial Dysfunction through Stabilization of PLIN5**

**Short Title: KIF13B Deficiency and Septic Cardiomyopathy**

Lianxin Zhang^1,#^, Guolin Miao^1,2,#^, Si Mei^1,#^, Yufei Han^1,#^, Yitong Xu^1^, Wenxi Zhang^1^, Jingxuan Chen^1^, Kaikai Lu^1^, Yinqi Zhao^1^, Zihao Zhou,^1^ Jinxuan Chen^1^, Jiabao Guo^1^, Pingping Lai^1^, Sin Man Lam^3,4^, Guanghou Shui^3^, Ling Zhang^1^, Weiguang Zhang^5^, Wei Huang^1^, Yuhui Wang^1^, Xunde Xian^1,5,6*^.

**Affiliations**

^1^ Institute of Cardiovascular Sciences, State Key Laboratory of Vascular Homeostasis and Remodeling, School of Basic Medical Sciences, Peking University, Beijing, China.

^2^ Department of Cardiology and Institute of Vascular Medicine, Peking University Third Hospital, Beijing, China.

^3^ State Key Laboratory of Molecular Developmental Biology, Institute of Genetics and Developmental Biology, Chinese Academy of Sciences, Beijing, China.

^4^ Lipidall Technologies Company Limited, Changzhou, 213022, Jiangsu Province, China.

^5^ Department of Human Anatomy and Histology and Embryology, School of Basic Medical Sciences, Peking University, Beijing, China

^6^ Beijing Key Laboratory of Cardiovascular Receptors Research, Peking University Third Hospital, Beijing, China

Correspondence to: Xunde Xian, PhD, Institute of Cardiovascular Sciences, State Key Laboratory of Vascular Homeostasis and Remodeling, School of Basic Medical Sciences, Peking University, No. 38 Xueyuan Rd, Haidian District, Beijing, China 100191. Email: xianxunde@bjmu.edu.cn.

^#^ These authors contributed equally to this work

* Corresponding authors.

**Supplementary Data**

**Supplementary figures and figure legends**

**Supplementary Figure 1. A mouse model of sepsis-induced cardiomyopathy is established using LPS or CLP stimulation.**

8-10 week-old male *WT* mice were injected with LPS (20 mg/kg) or perform cecal ligation and puncture (CLP) surgery (n=6/group).

**(A and D)** Quantitative analysis of plasma lactate dehydrogenase (LDH) from mice.

**(B and E)** Representative echocardiography M-mode images obtained from mice at selected intervals 6 h after LPS administration or 24 h after CLP surgery.

**(C and F)** EF%, FS%, LVESV, LVEDV, LVIDs and LVIDd were quantified via echocardiography (n=6/group).

Data were presented as mean ± SD and analyzed by unpaired student t-test in **A**, **C**, **D, F**.

**Supplementary Figure 2. Loss of Kif13b does not affect heart weight and ventricular dimensions in mice**

8-10 week-old male *Kif13b^-/-^* mice were injected with PBS or LPS (20mg/kg). (n = 6/group)

**(A)** The ratio of heart weight/Body weight (left) and heart weight/tibial length (right) in 8-week-old male *WT* and *Kif13b^-/-^* mice (n = 6/group).

**(B)** Representative images of HE staining of heart sections from mice. Scale bars, 2 mm for HE.

Data were presented as mean ± SD and analyzed by two-way ANOVA in **A.**

**Supplementary Figure 3. KIF13B deletion aggravates cardiac dysfunction and impairs cardiac metabolic homeostasis in cecal ligation and puncture-induced sepsis.**

8-10 week-old male *WT* and *Kif13b^-/-^* mice were perform CLP surgery (n = 4/group).

**(A)** Quantitative analysis of plasma LDH from mice.

**(B)** Representative echocardiography M-mode images obtained from mice at selected intervals (12h) after CLP surgery.

**(C)** EF%, FS%, LVESV, LVEDV, LVIDs and LVIDd were quantified via echocardiography (n = 4/group).

**(D)** Representative images and quantitative analysis of Masson, ORO, and 4HNE staining of heart sections from the indicated mice. Scale bars, 50 μm for Masson and ORO staining, 100 μm for 4HNE.

**(E)** Heart TG content (n = 3/group).

Data were presented as mean ± SD and analyzed by two-way ANOVA in **A**, **C**, **D** and unpaired student t-test in **E**.

**Supplementary Figure 4. Knockdown of KIF13B does not affect palmitoylation of CD36**

NRCMs were transfected with *scramble* or *siKif13b* and then stimulated with PBS or LPS (10 μg/mL) for 6 h. CD36 palmitoylation (Palm-CD36) in the presence or absence of hydroxylamine (HAM) treatment was detected using an immunoprecipitation and acyl-biotin exchange (ABE) (IP–ABE) assay. PD, pulldown.

**Supplementary Figure 5. Deficiency of KIF13B do not disrupt conversion of fatty acids to acyl CoA in the cardiac tissues of mice**

**A** qPCR analysis of mRNA expression in 8-week-old male WT and Kif13b-/- mice heart tissues (n = 6/group). **B** Heatmap of acetyl carnitine quantitative value in mouse heart tissue (n = 6/group) from metabolomics data. Data were presented as mean ± SD and analyzed by two-way ANOVA in **A**.

**Supplementary Figure 6. KIF13B confers cardioprotection via preventing PLIN5 deficiency-mediated mitochondrial dysfunction.**

**(A)** Protein abundances of PLIN5 in NRCMs transfected with *Scramble* or *siKif13b* (n = 3/group).

**(B-C)** NRCMs stained with 5 μM JC-1. Cells were excited at 555 or 488 nm and imaged by a confocal microscope. Scale bars, 100μm for JC-1 stanning (n = 6/group).

Data were presented as mean ± SD and analyzed by two-way ANOVA in **A** and one-way ANOVA in **B**, **C**.

**Supplementary Tables**

**Supplementary Table 1 gene siRNA sequence**

| Gene name | Sequences | |
| --- | --- | --- |
|  | Sense (5’ -3’) | Anti-sense (5’ -3’) |
| Human-*KIF13B* | CCUCCAUGAAGAACGAGAAUATT | UAUUCUCGUUCUUCAUGGAGGTT |
| Human-*KIF13B* | CCCAGUAAUACGAUCAUACUUTT | AAGUAUGAUCGUAUUACUGGGTT |
| Human-*KIF13B* | GCCUUGAAGAUCUGCGACAAATT | UUUGUCGCAGAUCUUCAAGGCTT |
| Rat-*Kif13b* | GCAGAAGCUUGUCAGUAAATT | UUUACUGACAAGCUUCUGCTT |
| Rat-*Kif13b* | GGACAGUUGCUGCAACCAATT | UUGGUUGCAGCAACUGUCCTT |
| Rat-*Kif13b* | GCAGCGAGUUCAGUGUUAATT | UUAACACUGAACUCGCUGCTT |
| Rat-*Plin5* | GCGCAGUGUGGAUGCUCUATT | UAGAGCAUCCACACUGCGCTT |
| Rat-*Plin5* | GGGACUAGACAAAUUGGAATT | UUCCAAUUUGUCUAGUCCCTT |
| Rat-*Plin5* | GCUGAACACUGUGUGUGUATT | UACACACACAGUGUUCAGCTT |

**Supplementary Table 2 Primer sequences for RT-qPCR**

| primers | Forward | Reverse |
| --- | --- | --- |
| mouse | | |
| *Gapdh* | CCAAGGTCATCCATGACAACTT | AGGGGCCATCCACAGTCTT |
| *Plin5* | GGTGAAGACACCACCCTAGC | CCACCACTCGATTCACCACA |
| *Kif13b* | GCTCTGTAGTGGACTCTTTGAAC | TTTGGGGTCAAGAAGGTCTCG |
| *Acad9* | TCCAGAGGTCAGTCAACATGA | CCTGGTCAATTTTTCGAGAGTCC |
| *Acadl* | AAACGTCTGGACTCCGGTTC | GTACCACCGTAGATCGGCTG |
| *Acads* | AAGTATGCCGAGAACCGCAA | CTGGATTTCGCTGGTCCCTT |
| *Abcd3* | GAGGAGCTTTACGGCCTGG | CTTATGCAGTGCCACGAACTT |
| *Acsl1* | AGTCTTTGCCACATCCGACC | AGTGCAAACCCAGTTGTGCT |
| *Acadvl* | TAGAAGCCGCCATCAGCAAA | AAACCACTGCCGATTCCTGT |
| *Hadha* | ACATCGGAGCTGTCTTTGGG | GACTCGTACTTCCGTAGCCG |
| *Hadhb* | TTCTGATTTCCCCACAGGCAG | CCGATGCAACAAACCCGAAA |
| rat | | |
| *Gapdh* | GACATGCCGCCTGGAGAAAC | AGCCCAGGATGCCCTTTAGT |
| *Plin5* | AGAGGCAGAAACAGGGCTAC | TTTGGGTGATGGAAAGTAGGGG |
| *Kif13b* | AGTGTTGGTGGAGGCTCAGTGG | GGCAAGCAGGCAGATGGTAGTG |

**Supplementary Table 3 peptide fraction separation liquid chromatography elution gradient table**

| Time (min) | Flow rate (mL/min) | mobile phase A (%) | mobile phase B (%) |
| --- | --- | --- | --- |
| 0 | 1 | 97 | 3 |
| 10 | 1 | 95 | 5 |
| 30 | 1 | 80 | 20 |
| 48 | 1 | 60 | 40 |
| 50 | 1 | 50 | 50 |
| 53 | 1 | 30 | 70 |
| 54 | 1 | 0 | 100 |

**Supplementary Methods**

**Western blot**

Total proteins from cultured cells or tissues were extracted using pre-cooled RIPA (DC302, DiNing), supplemented with a protease inhibitor (4693116001, Roche) and a phosphatase inhibitor (4906837001, Roche). Protein concentrations were quantified using a BCA protein assay kit (23225, Thermo Fisher Scientific). The lysates were mixed with supersampling buffer (P0015L, Beyotime) and denatured at 95 °C for 10 min, followed by SDS-PAGE and nitrocellulose membrane transfer. The membranes were immersed in 5% non-fat milk in TBST buffer (25 mM Tris, 137 mM NaCI, 2.7 mM KCI, 0. 075% Tween-20) for 1 h at room temperature (RT) and then incubated with anti-KIF13B (SAB2101257, Sigma), PLIN5 (26951-1-AP, Proteintech), CD36 (18836-1-AP, Proteintech), FABP3 (A01734-1, Boster), FATP1 (TD7716, Abmart), MT-COL(Mitochondrial Complex IV) (BA2149, Boster), UQCRC2(Mitochondrial Complex III) (14742-1-AP, Proteintech), SDHA(Mitochondrial Complex II) (14865-1-AP, Proteintech), NDVFS3 (Mitochondrial Complex I) (15066-1-AP, Proteintech), GFP (M20004, Abmart), HA (C29F4, Cell Signaling Technology) and GAPDH (60004-1-lg, Proteintech) primary antibodies were incubated overnight at 4°C. Afterward, the membrane was then washed three times with TBST and incubated with indicated horseradish peroxidase-conjugated secondary antibody (ZSGB-BIO, China) in TBST buffer supplemented with 5% BSA at RT for 1h. The target protein bands were visualised with enhanced chemiluminescence solution (36208ES, Yeasen) using the iBright Imaging System (CL1500, Invitrogen).

**Immunofluorescent staining**

Frozen heart sections (7 μm thick) or Paste cell crawls were fixed with 4% paraformaldehyde (PFA) for 15 mins at RT. The sections were followed by permeabilization with 0.2% Triton X-100 for 10 mins at RT. After blocking with 10% donkey serum in PBS for 1 hour at RT, sections were incubated overnight at 4°C with the following primary antibodies (all diluted 1:200 in blocking buffer): KIF13B (SAB2101257; Sigma-Aldrich), 4-HNE (ab48506, Abcam, USA). LAMP1 (553792, BD Biosciences), PLIN5 (26951-1-AP, Proteintech), GFP (M20004, Abmart), Tomm20 (A27800, ABclonal), HA (003-301-001, AlpVHHs) at 4°C, respectively. Normal isotype IgG (Cell Signaling Technology) was used as a negative control. After a rinse with PBS for 3 times, Alexa Fluor 488/555/647-conjugated secondary antibodies (ab150062, Abcam; ab150107, Abcam; ab150105, Abcam; ab150155, Abcam) were incubated in the dark at 37 °C for 1 h. Finally, 4',6-diamidino-2-phenylindole (DAPI, C1005, Beyotime, China) or Bodipy (D3922, Invitrogen, USA) were also used to display nuclei and lipid droplets, respectively. Super-resolution fluorescence imaging was performed by an integrated fluorescence microscope BZ-X810 (KEYENCE) or a confocal microscope FV3000 (Olympus). Immunofluorescence staining was quantified using Image J.

For reactive oxygen species (ROS) assay, after being washed with PBS for 3 times, cells were incubated with opti-MEM added containing 10 μM DCFH-DA (S0033S, Beyotime, China) at 37 °C for 30 mins. Super-resolution fluorescence imaging was performed by an integrated fluorescence microscope BZ-X810 (KEYENCE). Immunofluorescence staining was quantified using Image J.

For JC-1 assay, after being washed with PBS for 3 times, cells were incubated with 5 μM JC-1 (C2006, Beyotime) at 37 °C for 20 mins. Then, cells were washed twice with ice-cold staining buffer and analyzed within 30 mins. CCCP (10 μM, 20 mins) was used as a depolarization control. Super-resolution fluorescence imaging was performed by an integrated fluorescence microscope DMI6000B (Leica).

**Blood collection and biochemical analysis**

Blood samples were taken from the retro-orbital plexus, and plasma was separated by centrifugation (4000 rpm/min, 10 min, 4 °C). The samples were stored at -80 °C for future analysis. Plasma lactate dehydrogenase (LDH) levels were measured using the commercially available kits (LDH0360, Gensource Co., Ltd, China).

**RNA interference**

Small interfering RNA (siRNA) targeting rat *Kif13b* and *Plin5* as well as scramble sequences were synthesized by Sangon Biotech (Shanghai, China). The specific siRNA sequences are listed in Supplementary Table 1. NRCMs were seeded in 6-well plates at a density of 2 × 10^6^ cells/well with Opti-MEM medium and transfected with siRNA using lipofectamine RNAiMAX (13778150, Invitrogen) as described by the manufacturer. Cells were harvested 72 h after transfection to analyze knockdown efficiency.

**Preparation of lentivirus and infection**

Using the calcium phosphate method, 293T cells were co-transfected with lentiviral vectors (15 μg) alongside packaging plasmids pMDLg/pRRE, RSV/Rev, and VSV-G (5 μg each). After 12-16 h incubation, medium was replaced. Viral supernatant collected 48h post-transfection, filtered through 0.45-μm filters (Millipore SLHP033RB) and then concentrated to 1×10⁸ TU via ultracentrifugation for subsequent *in vivo/in vitro* applications. Lentiviruses were stored at -80 °C for future use.

Cells were infected with lentiviruses along with 1‰ polybrene (MC032, M&C Gene Technology, China) for 24 h. After 24 h infection, the culture medium was replaced with complete medium containing 1 ‰ doxycycline (T1687L, TargetMol). Then, cells were harvested 72 h after transfection to analyze overexpression efficiency.

**Plasmid construction**

The plasmids were constructed by Sangon Biotech (Shanghai, China). Cells were seeded on a 6-well plate with Opti-MEM medium and transfected with plasmids using Lipo3000 (Thermo Fisher Scientific, USA) as described by the manufacturer and then were harvested 48 hours after transfection for the analysis of the overexpression efficiency.

**Real-time quantitative polymerase chain reaction (qRT-PCR)**

Total RNA was extracted from tissues or cells using Trizol reagent (ET111-01- V2, TransGen) and reverse-transcribed to cDNA using the kit (AT321-01, TransGen, China). Gene expression levels were measured by mixing target primers with Top Green qPCR SuperMix (AQ132-24, TransGen), and the reactions were performed with QuantStudio 3 instrument (Thermo Fisher Scientific). Glyceraldehyde-3-phosphate dehydrogenase (GAPDH) was used as a housekeeping gene. Primer information is provided in Supplementary Table 2.

**Masson staining**

Paraffin-embedded tissue samples were sectioned, dewaxed, and dehydrated. Sections were stained with Weigert’s iron hematoxylin (5 mins), rinsed, treated with 1% phosphomolybdic acid (5 mins), and counterstained with 2% aniline blue (10 mins). Collagen fibers (blue), cytoplasm and muscle fibers (red) and nuclei (dark brown/black) were visualized. Fibrosis area was quantified using ImageJ v1.53 and expressed as % total tissue area. Masson’s trichrome staining was performed to quantify collagen deposition in heart tissues, indicative of fibrosis severity.

**Oil red O (ORO) staining**

Frozen heart samples embedded in optimal cutting temperature compound (4583, Sakura, USA) were sectioned at 7 μm, washed with 60% isopropanol for 10 s, and then stained with 0.3% ORO for 30 min, followed by counterstaining with hematoxylin for 3 mins.

**Hematoxylin and eosin (HE) staining**

Cardiac tissues from the designated experimental groups were harvested and fixed by immersion in 4% PFA overnight at 4°C. Following fixation, tissues were cryoprotected in a 20% sucrose solution for 24 h and subsequently embedded in paraffin. Sections of 5 μm thickness were cut and mounted on slides for standard hematoxylin and eosin (HE, Sigma, HHS128-4L, HT110232-1L) staining to evaluate general tissue morphology and structure.

**Triglyceride and NEFA assay**

The contents of triglyceride (TG) and free fatty acids (FFA) were measured in NRCMs using Triglyceride Assay kit (E1013, Applygen) and Non-esterified fatty acid (NEFA) kit (633-52001, Wako, Japan), respectively, following the manufacturers’ instructions.

**Co-immunoprecipitation (Co-IP) assay**

Cells were lysed in 1 ml RIPA buffer on ice for 30 mins. After vortexing for 15 s, lysates were centrifuged at 4 °C for 30 mins at 12,000 rpm/min. 80 μl of supernatants were mixed with 20 μl 5× loading buffer as an input sample. 800 μl of supernatants were incubated with 10 μl anti-GFP or anti- HA antibody at 4 °C for 6 h. Conjugated protein A/G agarose beads were added overnight at 4 °C. After being washed in lysis buffer for 5 times at 4 °C, beads were boiled with 120 μl 2× loading buffer at 95 °C for 5 mins and then centrifuged at 12,000 rpm/min for 1 min. The supernatants were used as pellet samples. Samples were analyzed by immunoblotting.

**Measurement of cellular oxygen consumption rate (OCR)**

The OCR was measured using the XF96 Analyzer (Seahorse Bioscience, USA). All procedures were carried out following the manufacturer’s guidelines. In summary, NRCMs were seeded at a density of 5 × 10^4^ cells/well of Seahorse XF96 cell culture microplate and incubated in low buffered non-bicarbonate assay medium (XF base medium with 2 mmol/L glutamine, 1 mmol/L sodium pyruvate, and 25 mmol/L glucose) for 1 h at 37 °C in a non-CO_2_ incubator before analysis using an XFe 96 extracellular flux analyzer (Seahorse Bioscience, USA). The rate of oxygen consumption was measured for 3 periods with a mixture of 3 mins in each cycle. Various inhibitors and activators were used at specific concentrations: oligomycin (1.5 μmol/L), carbonyl cyanide 4-(trifluoromethoxy) phenylhydrazone (FCCP, 1.5 μmol/L), antimycin A and rotenone (1.5 μmol/L). By utilizing these agents, the mitochondrial OCR was determined in different states including basal respiration, ATP production, and maximal respiration.

**Lipid extraction**

Lipids were extracted from approximate 30 mg of frozen tissues using a modified version of the Bligh and Dyer's method. Briefly, tissues were homogenized in 900 µl of chloroform: methanol: MilliQ H_2_O (3:6:1) (v/v/v). The homogenate was then incubated at 1500 rpm for 30 mins at 4 °C. At the end of the incubation, 350 µl of deionized water and 300 µl of chloroform were added to induce phase separation. The samples were then centrifuged and the lower organic phase containing lipids was extracted into a clean tube. Lipid extraction was repeated once by adding 500 µl of chloroform to the remaining aqueous phase, and the lipid extracts were pooled into a single tube and dried in the SpeedVac under OH mode.

**Molecular docking**

Rigid protein-protein docking of KIF13B and PLIN5 was performed using HDOCK SERVER (http://hdock.phys.hust.edu.cn/) to investigate their interaction. The protein structures of KIF13B and PLIN5 were obtained from the Protein Data Bank (PDB) (http://www.rcsb.org/). Protein interactions and subsequent visualization analyses were carried out using PyMOL (Version 2.4) and PDBePISA (https://www.ebi.ac.uk/pdbe/pisa/).

**Cardiac lipid analyses and lipidomics**

Lipidomic analyses were conducted at LipidALL Technologies using a Shimadzu Nexera 20-AD coupled with Sciex QTRAP 6500 PLUS. Separation of individual lipid classes of polar lipids by normal phase (NP)-HPLC was carried out using a TUP-HB silica column (i.d. 150x2.1 mm, 3 µm) with the following conditions: mobile phase A (chloroform: methanol:ammonium hydroxide, 89.5:10:0.5) and mobile phase B (chloroform:methanol:ammonium hydroxide:water, 55:39:0.5:5.5). MRM transitions were set up for comparative analysis of various polar lipids. Individual lipid species were quantified by referencing to spiked internal standards. d_9_-PC32:0(16:0/16:0), d_9_-PC36:1p(18:0p/18:1), d_7_-PE33:1(15:0/18:1), d_9_-PE36:1p(18:0p/18:1), d_31_-PS(d31-16:0/18:1), d_7_-PA33:1(15:0/18:1), d_7_-PG33:1(15:0/18:1), d_7_-PI33:1(15:0/18:1), C17-SL, d_5_-CL72:8(18:2)4, Cer d18:1/15:0-d_7_, d_8_-SM d18:1/18:1, C8-GluCer, C8-GalCer, d_3_-LacCer d18:1/16:0, Gb3 d18:1/17:0, d_7_-LPC18:1, d7-LPE18:1, C17-LPI, C17-LPA, C17-LPS, C17-LPG, d17:1 Sph, d17:1 S1P, C14-BMP, d3-16:0-carnitine were obtained from Avanti Polar Lipids. GM3-d18:1/18:0-d3 was purchased from Matreya LLC. Free fatty acids were quantitated using d31-16:0 (Sigma-Aldrich) and d8-20:4 (Cayman Chemicals).

Glycerol lipids including diacylglycerols (DAG) and triacylglycerols (TAG) were quantified using a modified version of reverse phase HPLC/MRM. Separation of neutral lipids were achieved on a Phenomenex Kinetex-C18 column (i.d. 4.6x100 mm, 2.6 µm) using an isocratic mobile phase containing chloroform:methanol:0.1 M ammonium acetate 100:100:4 (v/v/v) at a flow rate of 300 µL for 10 min. Levels of short-, medium-, and long-chain TAGs were calculated by referencing to spiked internal standards of TAG(14:0)3-d5,TAG(16:0)3-d5 and TAG(18:0)3-d5 obtained from CDN isotopes, respectively. DAGs were quantified using DAG-d5 (1,3-17:0) and DAG-d5 (1,3-18:1) as internal standards (Avanti Polar Lipids).

**Cardiac mRNA analyses and transcriptome**

RNA extraction

Total RNA was extracted from the tissue using TRIzol® Reagent according to the manufacturer's instructions. Then RNA quality was determined by 5300 Bioanalyser (Agilent) and quantified using the ND-2000 (NanoDrop Technologies). Only high-quality RNA samples were used to construct sequencing.

Library preparation and sequencing

RNA purification, reverse transcription, library construction and sequencing were performed at Shanghai Majorbio Bio-pharm Biotechnology Co., Ltd. (Shanghai, China) according to the manufacturer's instructions. The RNA-seq transcriptome librariy was prepared following Illumina® Stranded mRNA Prep, Ligation (San Diego, CA) using 1ug of total RNA. Shortly, messenger RNA was isolated according to polyA selection method by oligo(dT) beads and then fragmented by fragmentation buffer firstly. Secondly double-stranded cDNA was synthesized with random hexamer primers. Then the synthesized cDNA was subjected to end-repair, phosphorylation and adapter addition according to library construction protocol. Libraries were selected for size of cDNA target fragments of 300-400bp use magnetic beads followed by PCR amplified for 10-15 PCR cycles. After quantified by Qubit 4.0, the sequencing library was performed on NovaSeq X Plus platform (PE150) using NovaSeq Reagent Kit.

Quality control and read mapping

The raw paired end reads were trimmed and quality controlled by fastp with default parameters. Then clean reads were separately aligned to reference genome with orientation mode using HISAT2 software. The mapped reads of each sample were assembled by StringTie in a reference-based approach.

Differential expression analysis and functional enrichment

To identify DEGs (differential expression genes) between two different samples, the expression level of each transcript was calculated according to the transcripts per million reads (TPM) method. RSEM was used to quantify gene abundances. Essentially, differential expression analysis was performed using the DESeq2 or DEGseq. DEGs with |log2FC|1 and FDR < 0.05(DESeq2) or FDR < 0.001(DEGseq) were considered to be significantly different expressed genes. In addition, functional-enrichment analysis including GO and KEGG were performed to identify which DEGs were significantly enriched in GO terms and metabolic pathways at Bonferroni-corrected P-value < 0.05 compared with the whole-transcriptome background. GO functional enrichment and KEGG pathway analysis were carried out by Goatools and Python scipy software, respectively.

Alternative Splice events Identification

All the alternative splice events that occurred in our sample were identified by using recently releases program rMATS. Only the isoforms that were similar to the reference or comprised novel splice junctions were considered, and the splicing differences were detected as exon inclusion, exclusion, alternative 5', 3', and intron retention events.

KEGG PATHWAY Enrichment Analysis, were analyzed on the online platform of Majorbio Cloud Platform (<https://cloud.majorbio.com/>).

**Cardiac protein analyses and proteomics**

Total protein extraction

Samples were ground individually in liquid nitrogen and lysed with SDT lysis buffer (containing 100Mm Nacl) and 1/100 volume of DTT, followed by 5 min of ultrasonication on ice. After reacting at 95°C for 8-15 min and ice-bath for 2min, the lysate was centrifuged at 12000 g for 15 min at 4°C. And the supernatant was alkylated with sufficient IAM for 1 h at room temperature in the dark. Then samples were completely mixed with 4-time volume of precooled acetone by vortexing and incubated at -20°C for at least 2 h. Samples were then centrifuged at 12000 g for 15 min at 4°C and the precipitation was collected. After washing with 1mL cold acetone, the pellet was dissolved by Dissolution Buffer (DB buffer).

Protein quality test

BSA standard protein solution was prepared according to the instructions of Bradford protein quantitative kit, with gradient concentration ranged from 0 to 0.5 g/L. BSA standard protein solutions and sample solutions with different dilution multiples were added into 96-well plate to fill up the volume to 20 μL, respectively. Each gradient was repeated three times. The plate was added 180 μL G250 dye solution quickly and placed at room temperature for 5 minutes, the absorbance at 595 nm was detected. The standard curve was drawn with the absorbance of standard protein solution and the protein concentration of the sample was calculated. 20 ug of the protein sample was loaded to 12% SDS-PAGE gel electrophoresis, wherein the concentrated gel was performed at 80 V for 20 min, and the separation gel was performed at 120 V for 90 min. The gel was stained by Coomassie brilliant blue R-250 and decolored until the bands were visualized clearly.

Trypsin treatment

Each protein sample was taken and the volume was made up to 100 μL with DB lysis buffer (8 M Urea, 100 mM TEAB, pH 8.5), trypsin and 100 mM TEAB buffer were added, sample was mixed and digested at 37 °C for 4 h. Then trypsin and CaClz were added digested overnight. Formic acid was mixed with digested sample, adjusted pH under 3, and centrifuged at 12000 g for 5 min at room temperature. The supernatant was slowly loaded to the C18 desalting column, washed with washing buffer (0.1% formic acid, 3% acetonitrile) 3 times, then added elution buffer (0.1% formic acid, 70% acetonitrile). The eluents of each sample were collected and lyophilized.

Separation of fractions (High-depth Quantification)

Mobile phase A (2% acetonitrile, adjusted pH to 10.0 using ammonium hydroxide) and B (98% acetonitrile, adjusted pH to 10.0 using ammonium hydroxide) were used to develop a gradient elution. The lyophilized powder was dissolved in solution A and centrifuged at 12,000 g for 10 min at room temperature. The sample was fractionated using a C18 column (Waters BEH C18, 4.6×250 mm, 5 μm) on a Rigol L3000 HPLC system, the column oven was set as 45°C. The detail of elution gradient was shown in Supplementary Table 3. The eluates were monitored at UV 214 nm, collected for a tube per minute and combined into 10 fractions finally. All fractions were dried under vacuum, and then, reconstituted in 0.1% (v/v) formic acid (FA) in water.

LC-MS/MS analysis (EASY-nLC^TM^ 1200 UHPLC-HFX)

UHPLC-MS/MS analyses were performed using an EASY-nLCTM 1200 UHPLC system (Thermo Fisher, Germany) coupled with a Q ExactiveTM HF-X mass spectrometer (Thermo Fisher, Germany) in Novogene Co., Ltd. (Beijing, China). Firstly, mobile phase A (100% water, 0.1% formic acid) and B solution (80% acetonitrile, 0.1% formic acid) were prepared. The lyophilized powder was dissolved in 10 μL of solution A, centrifuged at 14,000 g for 20 min at 4 °C, and 1 ug of the supernatant was injected into a home-made C18 Nano-Trap column (4.5 cm×75 um, 3 um). The temperature of the column oven was set to 55 °C. Peptides were separated in a home-made analytical column (15 cm×150 µm, 1.9 µm), using a linear gradient elution as listed in Table 3.

The separated peptides were analyzed by Q ExactiveTM HF-X mass spectrometer, with ion source of Nanospray FlexTM(ESI), spray voltage of 2.1 kV and ion transport capillary temperature of 320°C. Full scan range from m/z 350 to 1500 with resolution of 60000 (at m/z 200), an automatic gain control (AGC) target value was 3×10^6^ and a maximum ion injection time was 20 ms. The top 40 precursors of the highest abundant in the full scan were selected and fragmented by higher energy collisional dissociation (HCD) and analyzed in MS/MS, where resolution was 15000 (at m/z 200), the automatic gain control (AGC) target value was 1×10^5^, the maximum ion injection time was 45 ms, a normalized collision energy was set as 27%, an intensity threshold was 2.2×10^4^, and the dynamic exclusion parameter was 20 s. The raw data of MS detection was named as ".raw".

Data analysis

The all resulting spectra were searched against Mus_musculus_uniprot_2022_9_5.fasta.fasta 86436 sequences) database by the search engines: Proteome Discoverer (Thermo, HFX and 480) or MaxQuant (Bruker, Tims).

The search parameters of Proteome Discoverer are set as follows: mass tolerance for precursor ion was 10 ppm and mass tolerance for product ion was 0.02 Da. Carbamidomethyl was specified as fixed modifications, Oxidation of methionine (M) was specified as dynamic modification, and loss of methionine at the N-Terminal. A maximum of 2 missed cleavage sites were allowed.

The search parameters of MaxQuant are set as follows: mass tolerance for precursor ion was 20 ppm and mass tolerance for product ion was 0.05 Da. Carbamidomethyl was specified as fixed modifications, Oxidation of methionine (M) was specified as dynamic modification, and acetylation was specified as N-Terminal modification. A maximum of 2 missed cleavage sites were allowed.

In order to improve the quality of analysis results, the software PD or MaxQuant further filtered the retrieval results: Peptide Spectrum Matches (PSMs) with a credibility of more than 99% was identified PSMs. The identified protein contains at least 1 unique peptide. The identified PSMs and protein were retained and performed with FDR no more than 1.0%. The protein quantitation results were statistically analyzed by T-test. The proteins whose quantitation significantly different between experimental and control groups, (P < 0.05 and [log_2_FC] > * (FC>* or FC < * [fold change, FC]), were defined as differentially expressed proteins (DEP).

The mass spectrometry proteomics data have been deposited to the ProteomeXchange Consortium (https://proteomecentral.proteomexchange.org) via the iProX partner repository with the dataset identifier PXD067683.

**ABE assay**

The ABE assay was performed to detect protein S-palmitoylation as described [50], with slight modifications. (1) Blocking: Cell lysates, prepared in lysis buffer (50 mM Tris-HCl, pH 7.5, 150 mM NaCl, 1 mM MgCl₂, 1% NP-40, 10% glycerol, protease inhibitor), were incubated with 50 mM N-Ethylmaleimide (NEM) (Solarbio, IE0880) for 2 h at 4°C to alkylate free thiols. (2) Enrichment & Cleavage: CD36 was immunoprecipitated from the lysates. The immunoprecipitates were then treated with 1 mM hydroxylamine (HAM) for 2 h at room temperature to specifically hydrolyze thioester-linked palmitate. (3) Biotinylation & Pull-down: Newly exposed cysteines were labeled with 5 mM biotin-HPDP (MCE, HY-136769) for 1.5 h at 4°C. After elution with 6 M urea, the samples were diluted and incubated overnight with streptavidin beads (MCE, HY-K0208) at 4°C. (4) Analysis: The beads were stringently washed with PBS containing 1% SDS, and the bound proteins were eluted in SDS sample buffer at 95°C for subsequent Western blotting.

**Untargeted Metabolomics**

1. metabolite extraction: Frozen tissue samples (100 mg) were pulverized in liquid nitrogen. The resulting powder was homogenized in pre-chilled 80% methanol, vigorously vortexed, and incubated on ice for 5 min. The homogenate was centrifuged at 15,000 × g for 20 min at 4°C. A portion of the supernatant was diluted with LC-MS grade water to a final methanol concentration of 53%, transferred to a new tube, and centrifuged again under the same conditions. The final supernatant was collected for LC-MS/MS analysis.
2. UHPLC-MS/MS analysis: Chromatographic separation was performed on a Vanquish UHPLC system (Thermo Fisher Scientific) equipped with a Hypersil Gold column (100 × 2.1 mm, 1.9 μm). A 17-min linear gradient was applied at a flow rate of 0.2 mL/min. The mobile phases consisted of 0.1% formic acid in water (A) and methanol (B) for positive ion mode, and 5 mM ammonium acetate (pH 9.0, A) and methanol (B) for negative ion mode. The gradient program was: 2% B (0–1.5 min), 2–85% B (1.5–4.5 min), 85–100% B (4.5–14.5 min), 100–2% B (14.5–14.6 min), and 2% B (14.6–17 min). Mass spectrometry was conducted on an Orbitrap Q Exactive HF mass spectrometer (Thermo Fisher Scientific) with electrospray ionization in both positive and negative modes. Key parameters included: spray voltage, 3.5 kV; capillary temperature, 320°C; sheath gas flow, 35 psi; auxiliary gas flow, 10 L/min; S-lens RF level, 60; and auxiliary gas heater temperature, 350°C.

(3) Data processing and metabolite identification: Raw data were processed using Compound Discoverer 3.1 for peak alignment, peak picking, and metabolite quantification. Parameters were set as: retention time tolerance, 0.2 min; mass tolerance, 5 ppm; signal intensity tolerance, 30%; signal-to-noise ratio, 3. Peak intensities were normalized to total spectral intensity. Molecular formulas were predicted based on additive ions, molecular ion peaks, and fragment ions. Metabolites were identified by querying the mzCloud, mzVault, and MassList databases. Statistical analysis was performed in R. Relative peak areas were calculated by normalizing sample values against quality control (QC) samples. Metabolites with a coefficient of variation (CV) > 30% in QC samples were excluded from further analysis. (4) Data analysis: Metabolites were annotated using the KEGG, HMDB, and LIPIDMaps databases. Principal component analysis (PCA) and partial least squares-discriminant analysis (PLS-DA) were conducted using the metaX platform. Differential metabolites were identified based on a variable importance in projection (VIP) > 1, p-value < 0.05 (Student’s t-test), and a fold change ≥ 2 or ≤ 0.5. Volcano plots were generated using the ggplot2 package in R. For clustering heatmaps, intensity values of differential metabolites were Z-score normalized and visualized using the Pheatmap package. Correlation analysis between differential metabolites was performed using Pearson’s method, with significance determined by cor.mtest and visualized using the corrplot package (p < 0.05). Functional and pathway enrichment analyses of differential metabolites were carried out based on the KEGG database. Enrichment significance was defined as p < 0.05.
