# Supplementary figures and images for: KIF13B Attenuates Sepsis-Induced Myocardial Dysfunction through the Stabilization of PLIN5
Source: Research (Wash D C). 2026 Jan 12;9:1033. doi: 10.34133/research.1033 (PMC12794201; doi:10.34133/research.1033)

**A**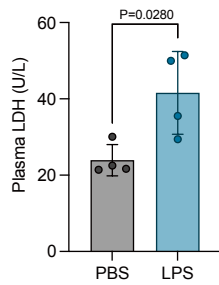**B**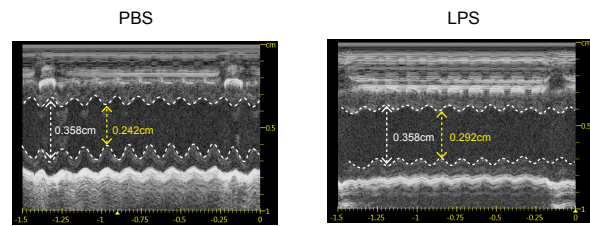**C**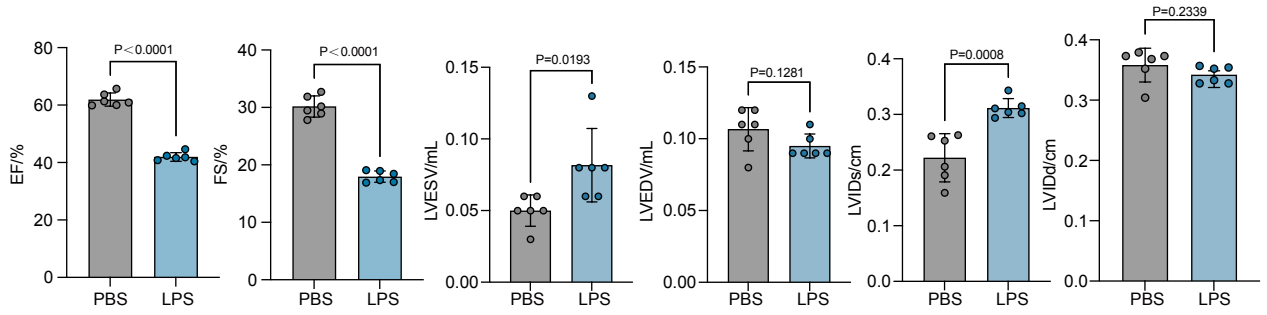**D**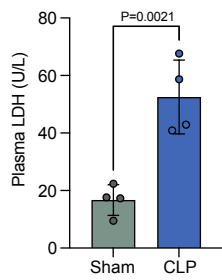**E**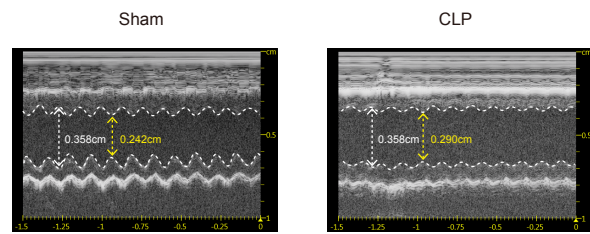**F**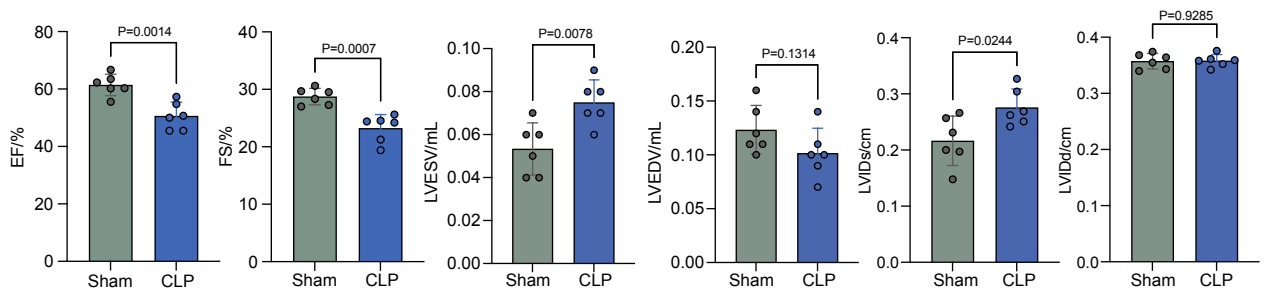

Supplement: Supplementary 1 — Supplementary Methods Figs. S1 to S6 Tables S1 to S3 Reference [50] [file research.1033.f1.zip › SP1.pdf]

**A**

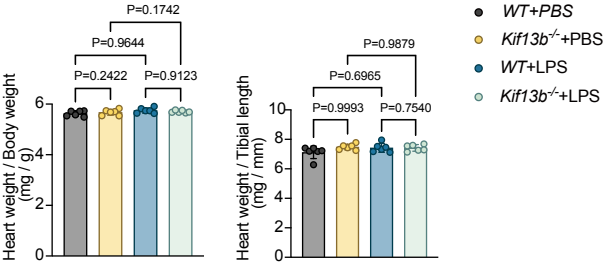

**B**

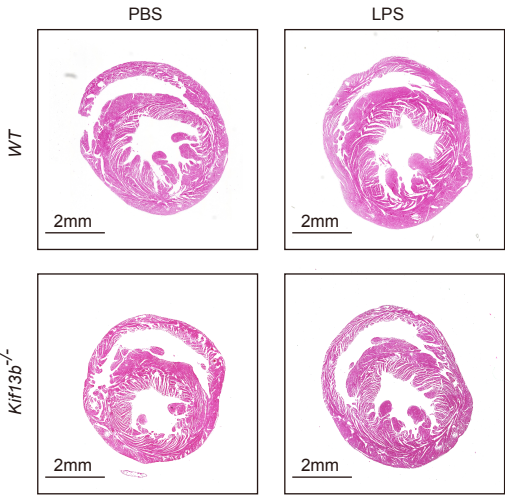

Supplement: Supplementary 1 — Supplementary Methods Figs. S1 to S6 Tables S1 to S3 Reference [50] [file research.1033.f1.zip › SP2.pdf]

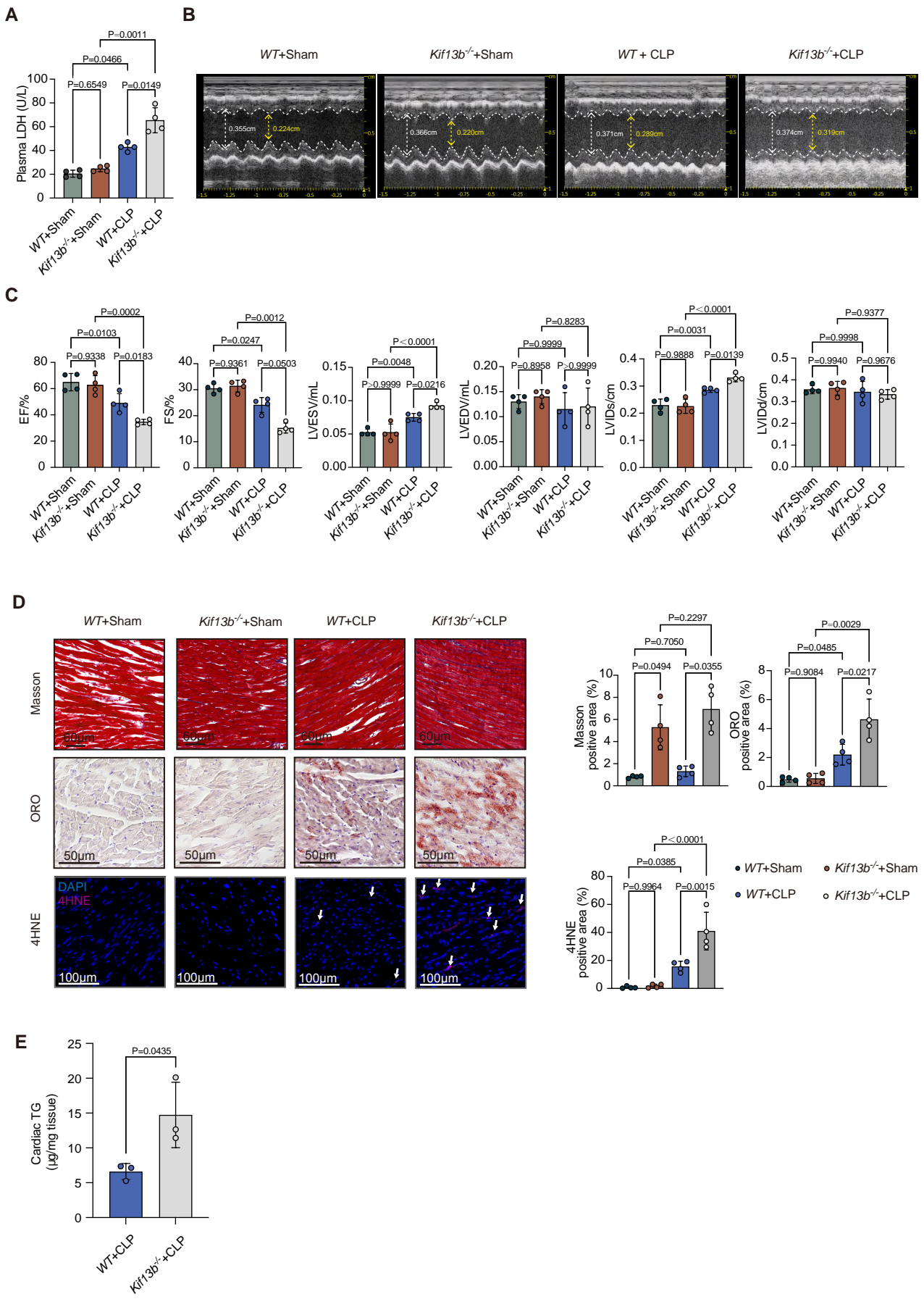

Supplement: Supplementary 1 — Supplementary Methods Figs. S1 to S6 Tables S1 to S3 Reference [50] [file research.1033.f1.zip › SP3.pdf]

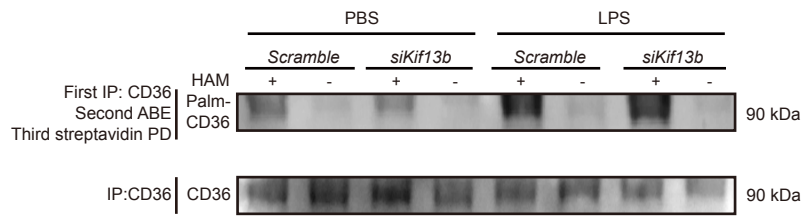

Supplement: Supplementary 1 — Supplementary Methods Figs. S1 to S6 Tables S1 to S3 Reference [50] [file research.1033.f1.zip › SP4.pdf]

● WT+PBS ● *Kif13b*<sup>-/-</sup>+PBS ● WT+LPS ● *Kif13b*<sup>-/-</sup>+LPS

**A**

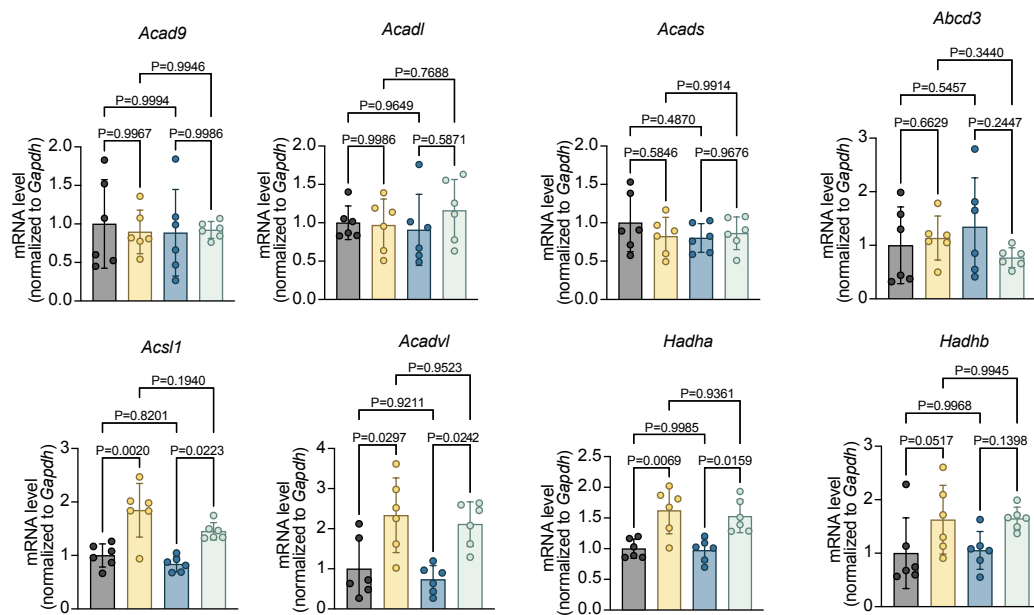

**B**

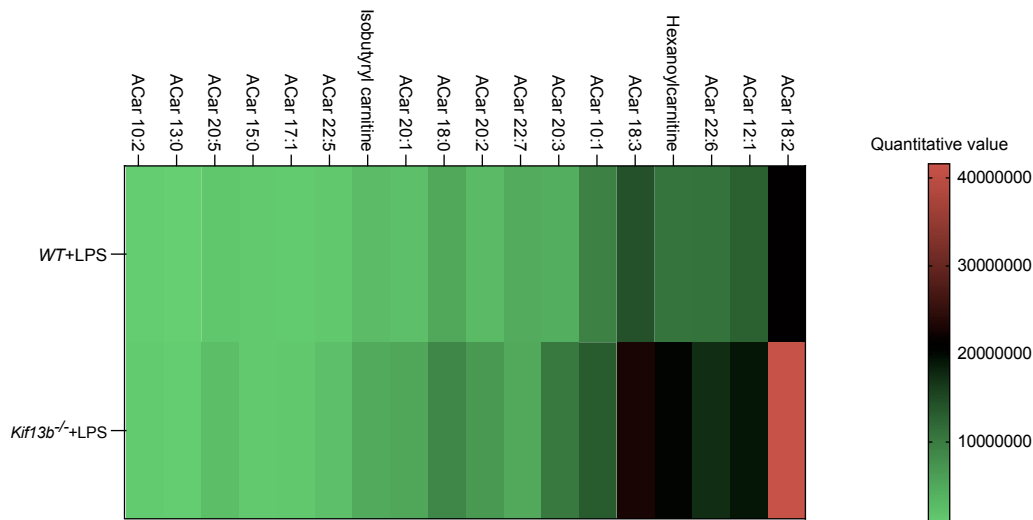

Supplement: Supplementary 1 — Supplementary Methods Figs. S1 to S6 Tables S1 to S3 Reference [50] [file research.1033.f1.zip › SP5.pdf]

**A**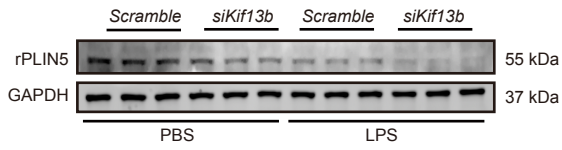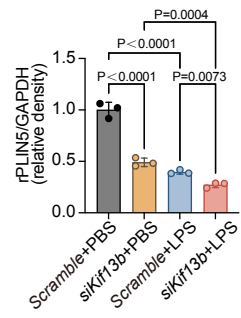**B**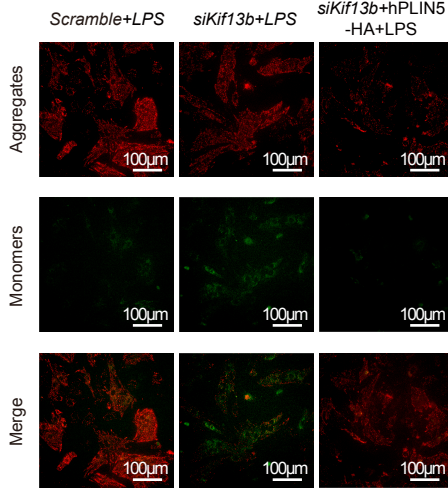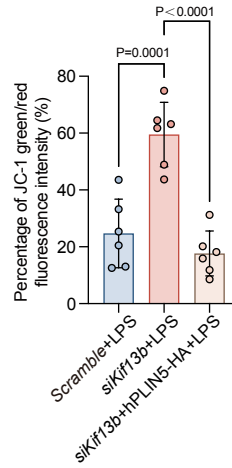**C**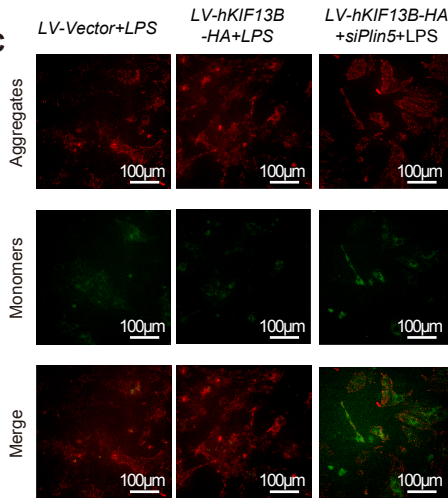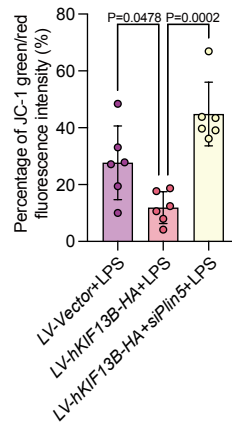

Supplement: Supplementary 1 — Supplementary Methods Figs. S1 to S6 Tables S1 to S3 Reference [50] [file research.1033.f1.zip › SP6.pdf]
